# Early Treatment Response in Black Smokers Undergoing Pharmacotherapy for Smoking Cessation: A Secondary Analysis of a Randomized Clinical Trial

**DOI:** 10.1001/jamanetworkopen.2023.34695

**Published:** 2023-09-20

**Authors:** Eleanor L. S. Leavens, Matthew S. Mayo, Alexandra R. Brown, Lisa Sanderson Cox, Edward F. Ellerbeck, Jasjit S. Ahluwalia, Nicole L. Nollen

**Affiliations:** 1 Department of Population Health, University of Kansas School of Medicine, Kansas City; 2 Department of Biostatistics and Data Science, University of Kansas School of Medicine, Kansas City; 3 Department of Behavioral and Social Sciences, Brown University School of Public Health, Providence, Rhode Island

## Abstract

This secondary analysis of a randomized clinical trial investigates the association of early treatment response with smoking cessation among Black smokers.

## Introduction

When smokers achieve abstinence in the first few weeks of treatment (ie, early treatment response), they have significantly greater odds of remaining abstinent at later times.^1^ Understanding factors that increase odds of early response may help identify which individuals are likely to have early success and could guide clinical decision-making. This analysis examined early treatment response (abstinence at 2 weeks) and its effect on long-term abstinence (at 26 weeks) and identified baseline factors that increased odds of early response.

## Methods

The parent study to this secondary analysis (not prespecified) was an open-label randomized clinical trial among smokers who self-identified as non-Hispanic Black and were interested in quitting smoking.^2,3^ After written informed consent, participants were randomized to standard (7 sessions of counseling with nicotine patch) or adaptive (including ≥2 pharmacotherapy adaptations [varenicline and bupropion with patch]) treatment. Groups received identical treatment with a 21-mg nicotine patch for the first 2 weeks. Adaptations occurred at weeks 2 and 6 based on biochemically confirmed smoking status, with carbon monoxide (CO) levels of 5 ppm or less indicating treatment response. Participants received 18 weeks of pharmacotherapy with long-term follow-up at week 26 (biochemically confirmed abstinence with anatabine and anabasine ≤2 ng/mL). The study was approved by the University of Kansas Medical Center’s Institutional Review Board and followed the CONSORT reporting guideline (see trial protocol in Supplement 1 and study flowchart in the eFigure in Supplement 2). This study did not require additional review, approval, or consent given that data were deidentified.

Table 1 presents baseline biopsychosocial variables selected for this analysis based on studies of factors that increased odds of cessation. The effect of early treatment response (week 2) on long-term abstinence (week 26) was assessed using logistic regression with treatment included as a factor in the model; multiple logistic regression using best subset variable selection was used to investigate factors that increased odds of early treatment response. For the best subset analysis, 2 modeling strategies were used. The first forced treatment into the model; the second allowed but did not force treatment into the model. Results were unchanged. The second modeling strategy was selected because all participants were on identical treatment through week 2. Analyses were conducted for week 2 and 26 completers only using SAS statistical software version 9.4 (SAS Institute). All tests were 2-sided, and statistical significance was set at .05.

**Table 1.** Baseline Factors and Their Association With Early Treatment Response^a^

Baseline factor	Participants, mean (SD) (N = 374)		*P* value^b^
	Responders (n = 129)	Nonresponders (n = 245)	
Demographic			
Age, y	54.9 (11.3)	51.9 (11.3)	.01
Socioeconomic			
Home ownership, No. (%)	27 (20.9)	37 (15.1)	.15
Household annual income, $	25 971.7 (22 704.7)	24 695.5 (19 268.9)	.59
Smoking characteristic			
CPD^c^	10.4 (5.9)	14.0 (7.3)	<.001
Menthol, No. (%)			.95
Yes	114 (88.4)	216 (88.2)	NA
No	15 (11.6)	29 (11.8)	NA
Cigarette craving^d^	32.1 (18.3)	34.1 (18.6)	.33
Nicotine withdrawal^e^	7.7 (6.3)	8.2 (6.5)	.52
Psychosocial			
Neighborhood problems^f^	14.9 (4.6)	15.0 (4.8)	.86
Neighborhood vigilance^g^	16.1 (5.4)	15.6 (5.4)	.39
Marijuana use, No. (%)^h^	26 (20.2)	59 (24.1)	.39
Other tobacco product use, No. (%)^i^	3 (2.3)	24 (9.8)	<.001
Perceived stress^j^	4.5 (3.4)	4.3 (3.4)	.61
Experience of discrimination^k^	5.9 (5.4)	5.7 (5.3)	.81
Resilience^l^	4.0 (0.8)	4.0 (0.8)	.61
Anxiety^m^	4.3 (4.9)	4.7 (5.5)	.43
Depression^n^	5.2 (5.3)	5.0 (5.0)	.96
Biological			
Cotinine level, ng/mL/1000	2.5 (1.9)	3.6 (2.8)	<.001
Nicotine metabolite ratio, ng/mL	2.9 (2.5)	3.8 (5.1)	.02

Abbreviations: CPD, cigarettes smoked per day; NA, not applicable.

^a^ Biochemically confirmed by carbon monoxide (CO) levels of 5 ppm or less at week 2.

^b^ *T* tests were performed for continuous measures, and χ^2^ tests were performed for categorical or dichotomous measures.

^c^ Participants self-reported number of CPD during the past 7 days.

^d^ Questionnaire of Smoking Urges-Brief (range, 10-70; higher scores indicate greater smoking urge).

^e^ Minnesota Tobacco Withdrawal Scale (range, 0-32; higher scores indicate greater symptoms of withdrawal).

^f^ A 10-item scale described problems that could arise in any area. Participants rated the extent of each problem using a 3-point scale from 1 (not a problem) to 2 (some problem) to 3 (serious problem). Answers were summed for the total score (range, 10-30).

^g^ Vigilance for Threat scale (range, 6-30; higher scores indicate greater perceived threat).

^h^ Participants self-reported marijuana use in the past 7 days (yes or no).

^i^ Self-reported past 7-day use of other tobacco products, including cigars, cigarillos, pipes, smokeless tobacco, hookah, or bidis (yes or no).

^j^ Perceived Stress Scale (range, 0-16; higher scores indicate greater perceived stress).

^k^ Frequency of perceived discrimination in day-to-day experience, with response options ranging from 0 (“never”) to 5 (“almost every day”); overall range was 0 to 25.

^l^ Brief Resilience Scale (range, 1-5; higher scores indicate greater resilience).

^m^ Generalized Anxiety Disorder-7 (0-4 [minimal anxiety], 5-9 [mild anxiety], 10-14 [moderate anxiety], and ≥15 [severe anxiety]).

^n^ Patient Health Questionnaire-2 (scores ≥3 may indicate major depressive disorder).

## Results

After accounting for treatment differences among 374 participants (mean [SD] age, 53.0 [11.4] years; 153 males [40.9%]), early treatment response increased odds of long-term abstinence (odds ratio, 3.6; 95 CI, 1.9-6.8; *P* < .001). Results were unchanged when individuals with missing data were treated as nonresponders (week 2) and smokers (week 26). See Table 1 for baseline factors for 129 early responders and 245 nonresponders. There were 3 factors that increased odds of early treatment response: fewer cigarettes per day, nonuse of other tobacco products, and lower cotinine levels (Table 2).

**Table 2.** Best 1-4 Factors Associated With Early Response^a^

Baseline factor	Early response, OR (95% CI)			
	Best 1 factor	Best 2 factor	Best 3 factor^b^	Best 4 factor
CPD^c^	0.91 (0.88-0.95)	0.91 (0.88-0.95)	0.93 (0.89-0.96)	0.93 (0.90-0.97)
Other tobacco use	NA	0.19 (0.06-0.65)	0.18 (0.05-0.63)	0.20 (0.06-0.70)
Cotinine level^d^	NA	NA	0.86 (0.78-0.96)	0.85 (0.76-0.94)
Nicotine metabolite ratio^e^	NA	NA	NA	0.93 (0.67-1.01)
Home ownership	NA	NA	NA	NA
Best model fit statistics	χ^2^, 22.0; AIC intercept covariates, 461.5; −2 log *L* intercept covariates, 457.5; proportion concordant, 63.1%; proportion discordant, 32.4%; *Somers D*, 0.31; γ, 0.32; τ-*a*, 0.14; *C* statistic, 0.65.	χ^2^, 29.8; AIC intercept covariates, 453.7; −2 log *L* intercept covariates, 447.7; proportion concordant, 65.6%; proportion discordant, 30.1%; Somers *D*, 0.36; γ, 0.37; τ-*a*, 0.16; *C* statistic, 0.68.	χ^2^, 37.1; AIC intercept covariates, 447.4; −2 log *L* intercept covariates, 439.4; proportion concordant, 69.4%; proportion discordant, 30.6%; Somers *D*, 0.39; γ, 0.39; τ-*a*, 0.18; *C* statistic, 0.69.	χ^2^, 40.6; AIC intercept covariates, 444.9; −2 log *L* intercept covariates, 434.9; proportion concordant, 70.0%; proportion discordant, 30.0%; Somers *D*, 0.40; γ, 0.40; τ-*a*, 0.18; *C* statistic, 0.70.

Abbreviations: AIC, Akaike information criterion; CPD, cigarettes per day; NA, not applicable; OR, odds ratio.

^a^ Early abstinence was defined by week 2 verified carbon monoxide (CO) levels of 5 ppm or less.

^b^ The 3-factor model had the best fit statistics while maintaining parsimony and significance of factors in the model.

^c^ Per 1-cigarette increase.

^d^ Per 1000-unit increase in cotinine.

^e^ Nicotine metabolite ratio is continuous.

## Discussion

In this study, early treatment responders had 3.6-fold greater odds of achieving long-term abstinence compared with nonresponders. Early responders smoked fewer cigarettes per day, reported nonuse of other tobacco products, and had lower cotinine levels at baseline. These findings may inform clinical decision-making by identifying patients likely to respond successfully to a nicotine patch, a minimal, low-cost intervention, and those who may benefit from more intensive or alternative intervention. Our findings underscore the importance of follow-up within 2 to 4 weeks of the quit date. If patients have not quit within 2 to 4 weeks, transitioning to a different treatment may be beneficial. Interestingly, the parent study showed that additional adaptations to other pharmacotherapies did not rescue a significant number of smokers.^3^ Consistent with the tipping point hypothesis, many smokers receive little benefit from even intensive interventions.^4^ For smokers unable to quit, harm reduction strategies, including e-cigarettes, are recommended as part of a shared decision-making approach.^5,6^

This study was limited to Black individuals who were light to moderate smokers with low prevalence of other tobacco use recruited from a single Midwestern city who were motivated to quit smoking. While this assessment included numerous biopsychosocial factors that may impact treatment response, other factors may merit future research.

## Notes

Supplement 1.
Trial Protocol

Supplement 2. **eFigure.** Study Flowchart

Supplement 3.
Data Sharing Statement

## References

[1] Ferguson SG, Gitchell JG, Shiffman S, Sembower MA. Prediction of abstinence at 10 weeks based on smoking status at 2 weeks during a quit attempt: secondary analysis of two parallel, 10-week, randomized, double-blind, placebo-controlled clinical trials of 21-mg nicotine patch in adult smokers. Clin Ther. 2009;31(9):1957-1965. doi:10.1016/j.clinthera.2009.08.029 19843485

[2] Nollen NL, Cox LS, Mayo MS, . Protocol from a randomized clinical trial of multiple pharmacotherapy adaptations based on treatment response in African Americans who smoke. Contemp Clin Trials Commun. 2022;30:101032. doi:10.1016/j.conctc.2022.101032 36387983 PMC9641174

[3] Nollen NL, Ahluwalia JS, Mayo MS, . Multiple pharmacotherapy adaptations for smoking cessation based on treatment response in Black adults who smoke: a randomized clinical trial. JAMA Netw Open. 2023;6(6):e2317895. doi:10.1001/jamanetworkopen.2023.17895 37338906 PMC10282892

[4] Baker TB, Bolt DM, Smith SS. Barriers to building more effective treatments: negative interactions amongst smoking intervention components. Clin Psychol Sci. 2021;9(6):995-1020. doi:10.1177/2167702621994551 35003904 PMC8740936

[5] Hartmann-Boyce J, McRobbie H, Butler AR, Electronic cigarettes for smoking cessation. Cochrane Database Syst Rev. 2021;9(9):CD010216. doi:10.1002/14651858.CD010216.pub6 34519354 PMC8438601

[6] Warner KE, Benowitz NL, McNeill A, Rigotti NA. Nicotine e-cigarettes as a tool for smoking cessation. Nat Med. 2023;29(3):520-524. doi:10.1038/s41591-022-02201-7 36788367

