# Supplementary material for: Early Treatment Response in Black Smokers Undergoing Pharmacotherapy for Smoking Cessation: A Secondary Analysis of a Randomized Clinical Trial
Source: JAMA Netw Open. 2023 Sep 20;6(9):e2334695. doi: 10.1001/jamanetworkopen.2023.34695 (PMC10512105; doi:10.1001/jamanetworkopen.2023.34695)
Supplement: Supplement 1. — Trial Protocol [file jamanetwopen-e2334695-s001.pdf]

University of Kansas Medical Center
RESEARCH PROTOCOL INVOLVING HUMAN SUBJECTS
TEMPLATE WITH GUIDANCE

Version date: September 23, 2021

Principal Investigator: Nicole Nollen

Study Title: Individualizing Pharmacotherapy: A novel optimization strategy to increase smoking cessation in the African American community

Co- Investigator(s): Lisa Sanderson Cox, Edward Ellerbeck, Matthew Mayo

I. Purpose, Background and Rationale

A. Aims and Hypotheses

African American (AA) smokers, who bear a disproportionate share of tobacco-related mortality, are poorly served by the one-shot tobacco treatment approaches that dominate research and clinical practice. Smoking cessation pharmacotherapy is effective for AA smokers but in general they achieve significantly lower absolute quit rates compared to non-AAs. A closer examination pinpoints early abstinence as an important but overlooked target for pre-emptive intervention. Abstinence rates within the first 4 weeks of treatment are 14%-36% for AA smokers compared to 34%-65% for non-AA smokers in studies testing the same pharmacotherapies. The low rate of early treatment efficacy in AA smokers is important because 75% of smokers who do not quit within 4 weeks of the target quit day (TQD) fail to achieve abstinence at the end of treatment and nearly all fail to achieve abstinence at later time points. Studies conducted among non-AA smokers have found that switching or augmenting pharmacotherapy for initial non-responders results in higher rates of abstinence. These studies examined the effects of altering pharmacotherapy only once after the TQD, yet in the treatment of other chronic diseases (e.g., diabetes, hypertension) it is common for providers to alter pharmacotherapy several times in order to achieve desired outcomes. Hence, for AA smokers there is a critical need to understand the effects of pharmacotherapy optimization to achieve early, and then sustained, abstinence. Moreover, for the field of tobacco treatment as a whole, there is further need to extend pharmacotherapy research to examine the effects of multiple medication alterations on early abstinence and long-term cessation.

The **long-term** goal of our research is to improve tobacco dependence treatment outcomes for AA smokers by identifying promising interventions. The **objective of this project** is to examine the efficacy of optimized (OPt) versus enhanced usual care (UC) treatment for smoking cessation. Optimization interventions have been widely used in other disciplines but are relatively new to tobacco dependence research. Given that tobacco dependence is a chronic, relapsing disorder characterized by multiple failed quit attempts, the high degree of heterogeneity in treatment response, and the absence of new pharmacotherapy advances, the best opportunities to improve tobacco treatment for AA smokers lie in the use of novel methodologies that explore how to optimize treatment through providing smoking cessation counseling while altering the timing, sequencing, or combining of existing pharmacotherapies to maximize efficacy. Consequently, AA smokers randomized to opt (n=196) will receive high intensity smoking cessation counseling, nicotine patch (NP), and up to two pharmacotherapy optimizations [bupropion (BUP), varenicline (VAR)] based on verified smoking status at Weeks 2 and 6. AA smokers randomized to enhanced UC (n=196) will receive the same high intensity counseling and NP with no optimizations in

pharmacotherapy. The **central hypothesis** is that OPT will result in significantly higher abstinence rates relative to UC.

Our specific aims are:

1) **Evaluate the short- and long-term efficacy of optimized pharmacotherapy for smoking cessation in AA smokers.** Hypothesis 1: Participants randomized to OPT will have significantly higher biochemically-verified abstinence at Week 12 (primary endpoint) than participants randomized to UC. Hypothesis 2: Participants randomized to OPT will have significantly higher biochemically-verified abstinence at two follow-ups, Week 18 and Week 26, than participants randomized to UC.
2) **(Mediators/Moderators): Identify mediators and moderators of the relationship between treatment (OPT, UC) and verified abstinence at week 12.** Hypothesis 3. Treatment engagement (medication use, counseling attendance), experience of side effects, and change in withdrawal, craving, reinforcing effects of nicotine, positive and negative affect, and self-efficacy to refrain from smoking will mediate the relationship between treatment and abstinence. Hypothesis 4. Gender, age, socioeconomic status (SES), nicotine exposure, menthol, and nicotine dependence will moderate the relationship between treatment and abstinence.
3) **(Process): Describe the response profile for each optimization pathway (OPT only).** There are 4 pharmacotherapy pathways that participants in OPT will potentially follow. We will describe the process of optimization including the proportion that follow each OPT pathway and demonstrate verified abstinence.

B. Background and Significance

African American (AA) smokers disproportionately impacted by tobacco. Tobacco remains the leading cause of preventable morbidity and mortality in the United States, accounting for more than 480,000 total deaths and more than 34% of all cancer death annually.[1] Although AA smoke fewer days per month, fewer cigarettes per day (CPD), and are less likely to be heavy smokers than Whites, they bear a disproportionate share of tobacco-attributable morbidity and mortality.[2] AA are at higher risk for nearly all smoking-related chronic diseases compared to Whites, have twice the rate of premature death attributable to cardiovascular disease, the highest incidence rates for all cancers combined, and higher overall cancer mortality rates compared to other racial/ethnic groups.[3,4] With regard to smoking-attributable lung cancer, specifically, AAs have a striking 43-55% higher relative risk compared to Whites with this difference being most pronounced at lower levels of daily cigarette consumption.[5]

AA smokers are less likely than White smokers to quit or respond to treatment. Quitting smoking is the single most important change a smoker can make to improve health,[6] yet AA are less likely to quit relative to non-AA despite being lighter smokers (< 10 cigarettes per day; CPD) and making more attempts.[7] According to 2015 national prevalence data, AA smokers are 16% more likely than White smokers to make a quit attempt in the past year but 31% less likely to achieve abstinence lasting > 6 months.[7] Differences persist in clinical trials where access and utilization of care are equalized.[8-12]

These findings have led to a misperception that smoking cessation pharmacotherapies are not efficacious for AA smokers. To the contrary, cessation pharmacotherapies have been found to be superior to placebo in RCTs conducted exclusively with AA smokers[13-15] and meta-analytic and review articles have consistently documented the efficacy of smoking cessation medications for AA smokers.[16-18] Based on the sum of evidence, *Clinical Practice Guidelines for Treating Tobacco Dependence* unequivocally recommend a combination of smoking cessation counseling and any one of the seven first-line medications - nicotine gum, nicotine patch (NP), lozenge, inhaler, nasal spray, bupropion SR (BUP), and varenicline (VAR) - to optimize cessation for all smokers, including African Americans.[6] The guidelines are also clear in the need for studies to improve cessation outcomes in racial/ethnic minority smokers. Our research team is one of a few in the U.S. with a line of research dedicated to improving tobacco dependence treatment for AA smokers. Our research spans two

decades and includes 5 NIH-funded pharmacotherapy trials of NP,[13] gum,[19] BUP,[14,15] and VAR[12] for AA smokers. While we have documented the efficacy of pharmacotherapy for AA smokers,[13-15] we have also noted a common theme in our work, and others,[8-11] specifically, that quit rates for AA smokers are lower than those found for non-racial/ethnic minority smokers on comparable therapies in the literature. Optimizing response to cessation therapy among AA smokers using an empirically-supported and novel adaptive treatment approach is the focus of this application. All participants will receive individualized, intensive smoking cessation counseling that has been developed in collaboration with an AA community advisory board and AA smokers over the past 15 years and has been found to be more effective than other counseling approaches with the target population.[19,20]

Why early treatment response? A closer look at the NIH-funded clinical trials conducted by our group illuminates early abstinence as an important, but overlooked, target for improving tobacco dependence treatment outcomes for AA smokers. Acknowledging the limitations of comparisons across studies, Table 1 shows biochemically-verified abstinence rates within the first 4 weeks of treatment for AA smokers in 4 out of 5 of our RCTs (early abstinence was not captured in our nicotine patch RCT[13]) and those achieved in major RCTs of the same therapies, which were conducted among predominately White smokers. As can be seen, 34-65% of non-AA smokers achieved early abstinence compared to 14-36% of AA smokers. Because efforts in AA have achieved moderate rates of early abstinence relative to non-minority smokers, concerted efforts are needed during this phase to improve short- and, subsequently, long-term treatment outcomes for AA smokers.

Table 1. Biochemically-confirmed abstinence rates within the first 4 weeks of treatment in RCTs among AA smokers and non-AA, predominately White smokers[a]

	Abstinence for AA	Abstinence for non-AA smokers
VAR	14% at Week 4[12]	42%-48% at Week 4[21,22]
BUP[b]	22% at Week 3[15]	60% at Week 4[23]
BUP[c]	36% at Week 1[14]	65% at Week 2[23]
	31% at Week 3[14]	60% at Week 4[23]
Nicotine gum	23% at Week 1[19]	34% at Week 4[24]

[a]Cessation not reported by race in these studies, therefore, the rates presented are for the total sample (79%-93% W); [b]AA light smokers (1-10 CPD); [c]AA heavy smokers (10+ CPD)

Early abstinence strongly predicts long-term treatment efficacy. Success within the first 4 weeks of initiating pharmacotherapy predicts long-term abstinence. Among those on NP who smoke within 2 weeks after beginning NP therapy, 66%-70% fail to achieve abstinence at the EOT (wks 6-10 depending on the study).[25-27] Among those on BUP, the combination of BUP + NP, or NP alone, only 7%-19% of those who smoked within 3 weeks of initiating pharmacotherapy were abstinent at the EOT compared to abstinence rates of 40%-67% for the entire sample.[26,28] Conversely, these same studies have found that 71%-80% of participants who responded (i.e., achieved abstinence) during the first 4 weeks of medication remained abstinent at the EOT.[25-27] We have found a similar pattern in our own work with AA. Among early responders (i.e., abstinent at Weeks 1 or 3), 78%-92% remain abstinent at the EOT and, of these, 54%-77% maintained long-term abstinence.[12,14,15] These data demonstrate the solid scientific premise that: 1) participants who do not achieve abstinence within 4 weeks of initiating medication are unlikely to achieve abstinence later; and 2) participants who do achieve early abstinence are likely to maintain abstinence through treatment and follow-up. Together, these findings support our scientific approach of continuing early responders on their existing pharmacotherapy but modifying pharmacotherapy for those not responding.

Lack of focus on early treatment non-responders is a missed opportunity. Many studies have focused on improving tobacco dependence treatment outcomes by preventing the return to smoking among responders. These relapse prevention studies assume a period of response/cessation and have had mixed success.[29] By comparison, few studies have focused on non-responders. If the majority of early treatment responders remain abstinent at the end of treatment and follow-up, focusing on this sub-group to the exclusion of treatment non-responders, who under the current treatment paradigm have a very low likelihood of achieving later abstinence, is a missed opportunity.

Lack of focus on non-responders is partly due to predominant use of study designs that do not allow for treatment adaptation.[30,31] A typical smoking cessation RCT randomizes participants at the start

of treatment and, while response to treatment is often monitored, re-assignment based on response rarely occurs. In addition, success in a standard cessation RCT is based on distal endpoints (e.g., Wk 26), with little attention paid to early endpoints (i.e., 2-4 weeks after treatment initiation) that are known to strongly predict long-term outcomes. Augmenting, switching, or stepping up treatment for non-responders could improve outcome but has rarely been explored within tobacco dependence research.[30,31]

C. Rationale

Scientific premise: Why an optimized therapy approach? Nicotine dependence is a chronic relapsing disorder characterized by multiple failed quit attempts.[1] The cyclical process of quitting smoking and the high level of between- and within-subject variability to treatment calls for a flexible treatment approach, yet typical cessation studies randomize participants at the start of treatment and rarely re-assign treatment based on response. To the contrary, optimization strategies that alter the type, dose, duration, frequency, and/or amount of treatment at critical, pre-defined decision points[32] are common place within other chronic diseases.[32-36] In hypertension and diabetes, for example, clinical trial protocols routinely incorporate modifications in pharmacotherapy based on blood pressure and glycemic response, respectively, and the success of adaptations have subsequently been incorporated into clinical practice recommendations. [37,38]

A growing body of cessation intervention studies have used an optimized therapy design, finding that optimization 'rescues' smokers who show poor initial response.[39-43] Of most relevance to the current study is the study by Rose et al. (2013) that assessed response to NP after 1 week of pre-cessation therapy and 1 week after the targeted quit date (TQD).[41] Responders at each time point remained on the NP, while non-responders were switched to either continuation of NP (control condition), 'rescue' treatment with BUP + NP, or 'rescue' treatment with VAR. Primary findings were that: 1) non-responders who were switched to BUP + NP or VAR had abstinence rates at the end of treatment and at 6 month follow-up that were ~10% higher than non-responders who remained on NP and 2) continuing the NP was highly effective among early responders. Another optimization study by Laude et al. (2017) treated smokers with 10 weeks of NP + BUP. At 10 weeks, responders either stopped medication completely or stayed on NP + BUP through week 26, while non-responders received VAR through week 26.[44] Week 26 cessation among week 10 responders was high (60% no meds, 60% NP + BUP) but also notable is that, among non-responders, late treatment optimization to VAR led to week 26 abstinence rates of 17% (up from 0% abstinence rates at week 10).

Non-intervention studies have also confirmed the benefit of optimization of smoking cessation medications. In a prospective cohort intervention study of 795 smokers using smoking cessation medications across multiple quit attempts, participants were classified as (1) early users (used medications for initial, but not subsequent quit attempts; (2) later users (used medications for subsequent, but not initial quit attempt); (3) repeaters (same medications used for each quit attempt); and (4) switchers (different medication used for each quit attempt).[45] Switchers had significantly higher week 4 abstinence rates relative to all other groups (28.5% versus 12.4%-20.0%) and, interestingly, the better quit rates among switchers (versus repeaters) was not accounted for by progression to a better cessation medication. Rather, the act of switching drove better success in quitting regardless of the medications smokers were switching to/from, providing solid scientific premise for our approach of switching versus extending (i.e., repeating) pharmacotherapy in non-responders.

The above studies support the strong scientific premise for an optimized approach to tobacco dependence treatment. Notably, 70-91% of participants in the above studies have been White smokers.[39-43] AAs have different smoking patterns and behaviors, including a preference for menthol cigarettes, fewer CPD, and different preferences for and responses to pharmacotherapy, including differences in nicotine and pharmacotherapy metobolims.[12,15,19,46-48] Therefore, the selection and sequence of smoking cessation medications

II. Research Plan and Design

A. Study Objectives

The objective of this study is to examine the efficacy of optimized (OPT) versus enhanced usual care (UC) treatment for smoking cessation. AA smokers randomized to OPT (n=196) will receive high intensity smoking cessation counseling, nicotine patch (NP), and up to two pharmacotherapy optimizations [bupropion (BUP), varenicline (VAR)] based on verified smoking status at Weeks 2 and 6. AA smokers randomized to enhanced UC (n=196) will receive the same high intensity counseling and NP with no optimizations in pharmacotherapy.

B. Study Type and Design

The proposed study is a randomized adaptive treatment trial for smoking cessation in 392 AA smokers. Participants randomized to OPT (n=196) will receive smoking cessation counseling, nicotine patch and up to two pharmacotherapy adaptations (VAR, BUP+NP) based on verified smoking status at Weeks 2 and 6. Participants randomized to UC (n=196) will receive the same smoking cessation counseling and NP; pharmacotherapy in this group will not be optimized. Pharmacotherapy and counseling in both groups will last for 18 weeks with long-term follow-up through Week 26. The primary outcome is biochemically-verified smoking status at Week 12.

C. Sample size, statistical methods, and power calculation

Sample size and power calculation. 392 eligible AA smokers will be randomized. We will use a 1:1 fashion to either OPT or enhanced UC. Randomization will be stratified based on to ensure an equal proportion of male and female smokers in OPT and UC. Randomization will be determined by computer-generated random numbers. Randomization assignments will be placed in sealed envelopes with sequential study ID numbers. After baseline data collection has been completed, the research assistant will select the sequential study ID number to determine the randomization assignment.

The primary endpoint is urine anatabine and anabasine-verified 7-day point prevalence abstinence at Wk 12. While CO is used for adaptation decision making, the more robust anatabine/anabasine are being used for biochemical verification of study endpoints, with the recommended cut-off of 2 ng/ml to differentiate smokers from non-smokers.60 These tobacco alkaloids are the gold standard for confirming abstinence when participants are on NRT and cotinine measurements are invalid because detectable levels of cotinine could reflect NRT use or smoking.60 We expect an 18% cessation rate at Wk 12 in the UC group and a 32% cessation rate in the OPT group. This difference in cessation is consistent with an adaptive trial which found that 10% of smokers who failed on one therapy and were switched to another early in treatment were rescued after one adaption.[35] Given two optimizations in the current study, we expect that ~15% of smokers will be rescued at Week 12 in OPT relative to UC. Using the chi-square test, along with 18% and 32% cessation rates, 196 participants in each group will give us 90% power to detect this difference with a type I error rate of 5%.

Statistical analysis for Aim 1 (abstinence): Aim 1 is the evaluate the short- and long-term efficacy of optimized pharmacotherapy for smoking cessation in AA who smoke. The hypothesis is that participants randomized to OPT will have significantly higher biochemically verified abstinence at week 12 (primary endpoint), week 18 (end of treatment), and week 26 relative to participants randomized to UC. Our primary analysis will be to compare the anatabine/anabasine-verified abstinence rate at week 12 between OPT and UC using the chi-square test. For our primary comparison, those lost to follow-up will be considered as smokers. We also will look at completers only and will utilize multiple imputation techniques to ensure valid comparisons between the two groups if the loss to follow-up is not random. We will also

compare the anatabine/anabasine-verified abstinence rates at weeks 18 and 24 between OPT and UC using the same methods described above. Secondarily, we will utilize Generalized Estimating Equation (GEE) methodology to longitudinally model verified abstinence at weeks 12, 18 and 24, examining treatment and mediators/moderators found in the best subset's logistic regression model (described below). These analyses will identify a final model utilizing the smallest subset of mediators/moderators that describes cessation over the length of the study.

Statistical Analysis for Aim 2 (mediation/moderation of treatment effect). If the treatment differences hypothesized in Aim 1 are found, we will explore the mediation and moderation hypothesized in Aim 2 using standard approaches. Aim 2 will identify mediators and moderators of the relationship between treatment and verified abstinence at week 12. The hypotheses are that treatment engagement, experience of side effects, withdrawal, craving, self-efficacy to refrain from smoking, and experience with the quitting process (e.g., cessation fatigue, thoughts about abstinence) will mediate the relationship between treatment and abstinence, while gender, age, socioeconomic status, menthol smoking, nicotine exposure and metabolism, and nicotine dependence will moderate the relationship between treatment and abstinence. For this type of modeling the number of events is more relevant than the total sample size. Assuming a total of ~100 confirmed quitters at week 12 (see sample size calculation above) and the general guideline of 10-30 events per factor in a model, we can expect to accommodate 3-10 factors (including treatment and intercept) in the model.

Statistical Analysis for Aim 3 (Describing optimization process; OPT only). Aim 3 will describe the response profile for each optimization path, including the proportion that follow each OPT pathway and demonstrate verified abstinence. Due to sample size, we are unable to do hypothesis testing between OPT pathways or test for subgroup differences (e.g., do participants in one path differ from participants in another path). However, in an exploratory manner, we will utilize extended or hidden Markov modeling techniques to help us understand and estimate the probabilities of success (cessation) down each path. Results of this model will give us the necessary estimates to design future studies comparing optimization strategies that could include varying the timing and number of optimizations.

D. Subject Criteria (See Vulnerable Populations appendix, if applicable)

Participants will be AA adults over the age of 18 who smoked cigarettes daily at enrollment and have smoked at this rate for ≥ 6 months. The study will be open to both men and women. We have chosen to focus on AAs only because their unique smoking profile places them at greater risk for tobacco-related disease and death.

Eligibility Criteria

Inclusion Criteria	Exclusion Criteria
• Non-Hispanic African American<br>• ≥ 18 years of age<br>• Smoke 5-30 CPD<br>• Daily cigarette smoker<br>• Smoked at current rate for ≥ 6 months<br>• Verified smoker (CO ≥ 5 ppm)<br>• Functioning telephone<br>• Interested in quitting smoking<br>• Willing to take NP, VAR , and/or BUP+NP for 18 weeks and complete all study visits	• Use of non-cigarette tobacco products in past 30 days<br>• Medical contraindications to NP, BUP, or VAR: unstable cardiac condition (e.g., unstable angina or AMI) cardiac event, or stroke in the past 4 weeks; renal impairment; medications contraindicted; history of clinically significant allergic reactions; history of epilepsy, seizure, head trauma, psychosis, bipolar disorder, eating disorder; unstable panic disorder or depression; active suicidal ideation; treatment for alcohol or drug dependence in the past year<br>• Use of pharmacotherapy in the month prior to enrollment<br>• Pregnant, contemplating getting pregnant, or breastfeeding<br>• Unstable housing (e.g., street, shelter)<br>• Plans to move from KC during the treatment and follow-up phase<br>• Another household member enrolled in the study

Inclusion criteria

Inclusion criteria are displayed in the adjoining table. Participants must smoke 5-30 CPD to be consistent with packaging guidelines for NP and minimize possible confounds in treatment response based on extreme light (1-4 CPD) or heavy (31+ CPD) smoking. The majority of AAs smoke 5-10 CPD and, combined, our CPD eligibility criteria encompasses ~85% of AA smokers.

Exclusion criteria

Exclusion criteria are also displayed in the adjoining table. Exclusion criteria are consistent with contra-indications for NP, BUP, and VAR. [13,14,86-93]. Non-daily smokers and those who have used non-cigarette tobacco products in the past 30 days (e.g., cigars, cigarillos) will be excluded because NP, BUP, and VAR have not been tested in these subgroups.

Withdrawal/Termination Criteria

There are no expected circumstances in which the subject's participation will be terminated by the investigator.

Clarify whether a study subject may participate in another research study while participating in this research study

Subjects are not able to participate in another smoking cessation research study while they are enrolled in the current study.

E. Specific methods and techniques used throughout the study

Laboratory tests

Not more than 20 ml of urine will be collected four times throughout the study. At Week 0 the urine will be used for the analysis of total nicotine equivalents (TNE) and other chemicals found in cigarettes. At Wks 12, 18 and 26 the urine samples will provide biochemical verification of **cessation** (via urine anabasine and anatabine, with the recommended cut-off of 2 ng/ml to

differentiate smokers from non-smokers). Not more than 20ml of saliva will be collected at Week 0 for genetic analysis. The DNA anaylsis will be used for research to understand nicotine metabolism genotype and phenotype. Melanin will be assessed at Wk 0 using a skin reflectometer.

Study Procedures:

Intervention

All eligible participants who provide written informed consent and complete the baseline survey will be randomized in a 1:1 fashion to either OPT or UC.

Optimization Overview. The optimization flow diagram is displayed in Figure 1. Both groups will receive 18 weeks of pharmacotherapy according to standard dosing guidelines [6,49] but the type of pharmacotherapy will vary depending on group assignment and response. **Path A** is participants randomized to enhanced UC who will receive 18 weeks of NP (Wks 0-18). **Path B1** is OPT participants who respond to NP (CO LTE 5 ppm) at each optimization time point and, therefore, remain on NP for 18 weeks (Wks 0-18). **B2** is OPT participants who respond to NP at Wk 2 but who demonstrate non-response to NP at Wk 6 (CO GT 5 ppm) and are switched to VAR. Participants in this group will receive 6 weeks of NP (Wks 0-6) and 12 weeks of VAR (Wks 6-18). **B3** is OPT participants who were switched to VAR at Wk 2 after non-response to NP and who demonstrate response to VAR at Wk 6. Participants in this group will receive 2 weeks of NP (Wks 0-2) and 16 weeks of VAR (Wks 2-18). **B4** is OPT participants who were switched to VAR at Wk 2 and BUP +NP at Wk 6. Participants in this group will receive 2 weeks of NP (Wks 0-2), 4 weeks of VAR (Wks 2-6), and 12 weeks of BUP +NP (Wks 6-18).

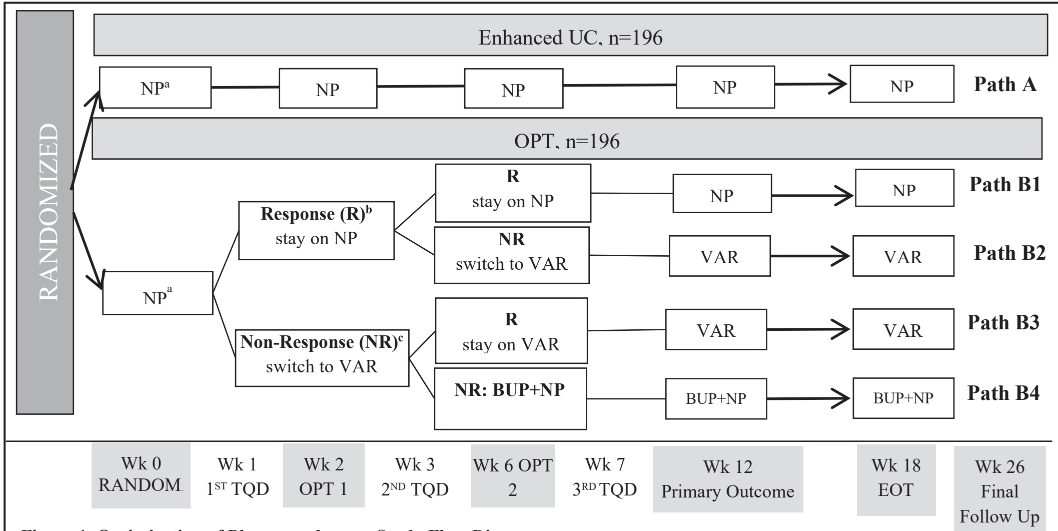

Figure 1. Optimization of Pharmacotherapy Study Flow Diagram
[a]NP = nicotine patch
[b,c] At each time point**, response (R)** defined as a CO LTE 5 ppm and **non-response (NR)** to defined as a CO GT 5 ppm

Use of study medication will be under the supervision of Drs. Nollen (PI), Ellerbeck (Co-I and Kansas study physician), and Salzman (Co-I and Missouri study physician). Participants will be prompted to discuss side effects at each in-person visit and given the study phone number to report adverse events at interim time points. NIH guidelines for reporting serious adverse events (SAEs) will be followed. Any problems needing medical attention will be referred to Drs. Ellerbeck or Salzman who will carry a study pager and have served in this role in previous RCTs. Medication will be discontinued or reduced following a SAE or in the event of AEs judged by the clinical team to warrant discontinuation or reduction. In our previous RCTs with these medications only 3% of participants required discontinuation and 1% required reduction.

NP (OPT, UC). To maximize efficacy, all participants will receive the 24-hour 21 mg NP for 18 weeks. NP therapy will start at Week 0, with the 1st TQD occurring at Week 1.

VAR (OPT). VAR will be dispensed 0.5 mg once daily on Days 1-3, 0.5 mg twice daily on Days 4-7, and 1 mg twice daily from Day 8 through the EOT. The new (2nd TQD will be Day 8 of VAR. Treatment with VAR has been approved for up to 26 weeks, with the standard course of treatment being 12 weeks.[50] Non-responders in the current study will receive VAR for 4 weeks (Path B4), 12 weeks (B2), or 16 weeks (B3).

BUP (OPT). BUP will be dispensed 150 mg once daily on Days 0-3 and then 150 mg twice daily from Day 4 through optimization or the EOT. The new (3rd) TQD will be Day 8 of BUP. Treatment with BUP has been approved for up to 12 months, with the standard course of treatment being 12 weeks.[50] Non-responders in the current study will use BUP +NP for 12 weeks (Path B4).

Counseling (OPT, UC). Both groups will receive 7 sessions of patient-centered solution-focused counseling for smoking cessation.[6] The counseling protocol is evidence-based,[6,51,52] individualized and culturally-specific, has been developed by our team over the last 20 years in collaboration with an AA community advisory board and former AA study participants,[20] and has been found to be superior for AA smokers in head-to-head comparisons with other counseling approaches (i.e., motivational interviewing, brief advice).[19,53] The goal of counseling is to increase knowledge (including information particularly relevant to AA), develop skills (including behavioral and cognitive strategies) based on individual needs, and provide intra-treatment social support. Skills include medication management and adherence, coping responses to address withdrawal/craving and lapses, strategies to address smoking triggers, reduce cue-smoking contingencies, and increase self-efficacy to promote abstinence and support a smoke-free lifestyle. Our approach is intentionally flexible to match the needs of each participant over the course of behavior change. Counseling goes hand-in-hand with a 30-page culturally-targeted Kick It at Swope Quit Smoking Guide, used in previous KIS trials. For the purposes of rapport and continuity of care, participants will meet with the same counselor each session. Counseling will be delivered by our experienced staff who are certified tobacco treatment specialists, have extensive experience treating AA smokers within clinical trials, and are active members of the community. Dr. Cox (co-I), a licensed clinical psychologist, will be responsible for counselor training and supervision. Time points for counseling have been selected to correspond with ongoing CO monitoring of smoking status/response to treatment (Wks 0, 2, 6, 12) and one week following optimization/assessment time points, which will coincide with TQDs (Wk 1 for all, and Wk 3, 7 for non-responders in both groups).

Procedures and Methods

An overview of major study events is provided in the adjoining table.

Initial Screening. The initial screen will review inclusion/exclusion criteria. Those eligible will be scheduled to complete final eligibility screening within 14 days. All ineligible smokers will be referred to local resources.

Final Screening and Enrollment (Wk 0). Final eligibility screening will be conducted in person and will consist of a pregnancy test on women of childbearing age and obtaining informed consent.

Counseling Visits (Wks 0, 1, 2, 3, 6, 7, 12). Counseling sessions, each lasting approximately 20 minutes, will be completed in person at Wks 0, 2, 6, 12 and by telephone at Wks 1, 3, 7. The number of sessions is consistent with our previous studies where we have achieved visit completion rates of ~ 80%.[12,14,15,19]

Response/Non-Response to Pharmacotherapy (Wk 2, 6). Exhaled air carbon monoxide (CO) is an immediate, non-invasive method of biochemically verifying smoking status. At Wk 2 and 6, all participants will breathe into a Micro-3 Smokerlyzer (Bedfont Scientific) for the purpose of determining abstinence. All participants will be informed of their CO level and it will be used to frame counseling discussions around smoking status/quitting. OPT participants with CO LTE 5 ppm will be considered 'responders' and will continue on their existing pharmacotherapy. Those with CO GT 5 ppm will be considered 'non-responders' and will be switched to the next pharmacotherapy per Figure 1.

Overview of Major Study Events and Measures

	Screen	Enroll						Primary Endpoint	EOT	Follow-Up
		WEEKS								
	-2*	0	1*	2	3*	6	7*	12	18	26
Screening	X	X								
Intervention										
Counseling		X	X	X	X	X	X	X		
Medication Dispensing		X		X		X		X		
Smoking Abstinence										
CO		X		X		X				
Urine for TNE & Anabasine/Anatabine		X						X	X	X
Saliva		X								
Mediators										
Withdrawal		X	X	X	X	X	X	X	X	
Craving		X	X	X	X	X	X	X	X	
Reinf effects of nicotine		X	X	X	X	X	X	X	X	
Pos & Neg Affect		X	X	X	X	X	X	X	X	
Self-efficacy		X	X	X	X	X	X	X	X	
Side effects			X	X	X	X	X	X	X	
Medication use			X	X	X	X	X	X	X	
Counseling completion		X	X	X	X	X	X	X		
Moderators (all assessed at baseline)										
See measures		X								
Process Measures (OPT only)										
# down each OPT path								X		
# reinitiating medication					X		X			
# setting new TQD					X		X			
Verified abstinence								X	X	X
Change in withdrawal, craving, reinf effects of nicotine, affect, and self-efficacy post-optimiz.				X	X	X	X	X		
Descriptive Measures (all assessed at baseline)										
See measures		X								
Visit Length (in minutes)	30	120	45	60	45	60	45	120	45	45

*phone visit; all other visits are in-person

Medication Dispensing (Wk 0, 2, 6, 12). Medication will be dispensed at Wk 0 (baseline) and Wk 2, 6, 12 in the quantity needed to get participants to the next assessment/optimization time point. This approach has resulted in 88% attendance at medication dispensing visits in our previous studies. Participants will be asked to return unused medication to ensure (a) that OPT participants take only the medication corresponding with the current stage of treatment and (b) that OPT and UC participants on NP receive a supply that corresponds with their current dosing needs. Those who do not attend medication dispensing visits will be mailed enough of their *current* medication to get them to the next dispensing time point. Participants must be eligible for NP, BUP, and VAR as a condition of eligibility; however, prior to switching, we will reassess medical eligibility in OPT non-responders. Given the short time frame between visits and careful upfront screening, we expect that very few non-responders will be medically ineligible for switching. Those who are will either receive the next therapy for which they are eligible (BUP, VAR) or, if medically ineligible for either switch, stay on their existing pharmacotherapy.

COVID-19 Remote Visits.

Beginning March 23, 2020 and for the duration of the "COVID-19 Stay at Home Order' all face-to-face encounters will be temporarily terminated. All previous in-person assessments will be completed over the phone. Urine samples will be either mailed in or left outside for pickup by study staff as determined by location and abilities of the study participant. Medication will be dispensed similarly; either by mail or dropped at the study participant's home. Instructions and counseling sessions will be completed by phone and/or facetime. Because enrollment activities cannot be completed remotely, study enrollment will be temporarily suspended.

When the 'Stay at Home Order' is lifted or COVID-19 precautions are no longer deemed necessary, the original study protocol will go into effect.

Retention. Retention is enhanced by having participants meet with the same counselor each time. We have also developed a system of reminders and compensation for participant efforts that have resulted in impressive retention rates in our previous clinical trials. **Reminders**. Five days prior to each visit a reminder postcard, email, and/or text noting the scheduled appointment date and time will be sent. Participants will also be called, texted, or emailed (based on preference) up to 6 times to remind them of their upcoming visit. A detailed tracking database, with an automated reminder system, will notify counselors of when to send reminder messages. **Compensation**. Participants will be given a $30 gift card at Wks 0, 2, 6 and 12, a $20 gift card at Wk 18, a $40 gift card at Wk26, and a $10 gift card at Wks 1, 3, 7 as compensation for their time/travel. Participants may receive an additional $20 at Wks 2, 6, 12 and 18 for returning their unused medication.

Clearly indicate which procedures, tests, visits, etc., are parts of usual standard therapy and which are performed solely for research purposes.

All tests, procedures, and visits are being performed solely for research purposes and are not billable to insurance companies.

Describe the fate of any body component (blood, CSF, bone marrow, etc.) used in the study, emphasizing confidentiality of labeling of the sample and the sample's destruction or storage.

Samples will be labeled only with a unique study identification number and only members of Dr. Nollen and Reed's team will have access to the samples. Samples will be disposed of one month after the final report is sent out to the Principal Investigator, unless participants agree to have their urine stored for future testing.

F. Risk/benefit assessment

Physical risk

Risks for participating are primarily those associated with the use of pharmacotherapy. Common side effects occurring in $\geq$5 % of patients is summarized below for each medication. To address these concerns, participants will receive written information and information from their counselor about how to manage any of these symptoms. Risks will be minimized by our extensive exclusion criteria which encompass contraindications for NP, VAR, and BUP.

Nicotine Patch:

- Sweating
- Redness or swelling at the patch site
- Irritability
- Problems sleeping or vivid dreams
- Headache
- Dizziness
- Nausea or vomiting
- Abnormal heartbeat or rhythm
- Diarrhea Nervousness or restlessness
- Difficulty breathing
- Tiredness

Bupropion and Varenicline Risks:

- Nausea
- Sleep problems (trouble sleeping or vivid, unusual or strange dreams)
- Headache
- Constipation
- Gas
- Dry mouth
- Dizziness
- Disturbance in attention
- Fatigue
- Skin changes (dry skin or acne)

Psychological risk

Risks for participants in both treatment arms (OPT, UC) also include those associated with the inconvenience of participation including answering surveys, providing blood and urine, and participating in follow-up visits and assessments. To minimize the inconveniences associated with study participation we will review all data collection instruments and study procedures to minimize the number of items in our instruments and improve the accessibility and convenience of our study procedures. We anticipate using several methods to enhance convenience to participants, including offering study visits in the evening and on weekends and offering patients a choice of where they will be seen (KUMC or Swope). Another risk is feeling pressured to be in the study, which we will track in order to monitor and will report this as an adverse event.

Social risk

None

Economic risk

None

Recall of Varenicline: September 2021:

Pfizer issued a voluntary recall of all lots of 0.5mg and 1mg tablets of varenicline on September 21,2021. At this point in the study, we had 17 enrolled participants remaining on adapted medication. Of these 17, 2 were assigned to varenicline; however, both had missed their previous medication refill and therefore did not have a supply of varenicline. They were not offered further adaptation as they are at the end of the medication treatment phase of the study (i.e., week 18). No additional varenicline will be dispensed in the study.

At the time of the recall, study enrollment was complete, and all medication dispensing was complete. Only follow-up of participants remained.

Potential benefit of participating in the study

There are also no direct benefits to participating in this study except that researchers hope that the information from this research study may be useful in improving treatment for AA smokers.

G. Location where study will be performed:

The study will take place at Swope Health Central, 3801 Blue Parkway, in Kansas, Missouri or at the KUMC CRU in Fairway, Kansas based on the preference of each participant. All data will be directly entered into an electronic data capture system (i.e., RedCap or CRIS), therefore minimizing the use of paper records. If paper records are generated, they will be stored in locked file cabinets at Swope. Only study staff will have access to the locked records at Swope and the secure online electronic data capture system.

H. Collaboration (with another institution, if applicable): Gary Salzman, M.D. will serve as a co-investigator and the PI of the subcontract to Truman Medical Center (TMC). TMC is one of the largest provider of safety net medical care for uninsured and under-insured patients in the Kansas City metropolitan are. Dr. Salzman is the Chief of Respiratory and Critical Care at TMC whose patient population is 40% African American. Dr. Salzman will lead recruitment efforts of TMC patients. He will participate in weekly team meetings and review documents as needed. KUMC will serve as the lead IRB.

I. Single IRB Review for a Multi-site study (if applicable): N/A

J. Community-Based Participatory Research (if applicable): N/A

K. Personnel who will conduct the study, including:

Indicate, by title, who will be present during study procedure(s)

Personnel on the project include: Nikki L. Nollen (PI), Lisa Cox (co-I), Ed Ellerbeck (co-I and study physician), Allen Greiner (co-I and Study physician), Matt Mayo (co-I, lead biostatistician), Tricia Snow (project director), Lexi Brown (senior research analyst), Genevieve Casey (research assistant), Brian Hernandez (research assistant), Terri Tapp (research assistant), and Michael Arnold (GRA).

1. Primary responsibility for the following activities, for example:

   a. *Determining eligibility:* Ed Ellerbeck, Allen Greiner, Nikki Nollen, Tricia Snow, , Brian Hernandez, Terri Tapp, Michael Arnold

   b. *Obtaining informed consent:* Tricia Snow, , Brian Hernandez, Terri Tapp, Michael Arnold

   c. *Providing on-going information to the study sponsor and the IRB:* Nikki Nollen, Tricia Snow

   d. *Maintaining participant's research records:* Tricia Snow, Brian Hernandez, Terri Tapp, Lexi Brown, Dinesh Mudaranthakam, Matt Mayo

   e. *Completing physical examination:* Not applicable

   f. **Taking vital signs, height, weight: Height and weight will be the only vital signs** taken. They will be performed by Tricia Snow, Brian Hernandez, Terri Tapp

   g. *Drawing / collecting laboratory specimens:* Tricia Snow, Brian Hernandez, Terri Tapp

   h. *Performing / conducting tests, procedures, interventions, questionnaires:* Tricia Snow, Brian Hernandez, Terri Tapp

   i. *Completing study data forms:* Tricia Snow, Brian Hernandez, Terri Tapp, Lexi Brown, Michael Arnold

   j. *Managing study database:* Matt Mayo, Lexi Brown, Dinesh Mudaranthakam, Tricia Snow

L. Assessment of Subject Safety and Development of a Data and Safety Monitoring Plan

The current study involves three FDA-approved smoking cessation medications -- nicotine patch (NP), bupropion (BUP), and varenicline (VAR). These medications do not require an investigational new drug application and are being used in accordance with cleared/approved labeling. Specifically, nicotine patch is available over-the-counter without a prescription and is approved for long-term use. Participants in the current study will use the nicotine patch for up to 18 weeks or for a shorter duration if it is determined that the nicotine patch is not working for them. Bupropion (BUP) and varenicline (VAR) both require a prescription. Participants will receive clearance from a physician to use these products before being enrolled and will be under the medical supervision of a physician during the course of the study. Treatment with VAR has been approved for up to 24 weeks, with the standard course of treatment being 12 weeks.[50] Participants in the current study will receive VAR for 4 weeks, 12 weeks, or 16 weeks. The shorter course of treatment (i.e., 4 weeks) will only occur if VARis not working for the participant (i.e., they are not quit and/or are experiencing substantial nicotine-related withdrawal or craving and medication-related adverse events). These are standard instances in which the use of VAR would be terminated. Treatment with BUP has been approved for up to 12 months, with the standard course of treatment lasting from 6 – 12 weeks.[50] Participants in the current study will use BUP for 12 weeks. The black box warning for both BUP and VAR has been removed (https://www.fda.gov/Drugs/DrugSafety/ucm532221.htm) due to evidence that these medications do not increase neuropsychiatric harm relative to placebo; this is true in individuals with and without a mental health diagnosis.[54]

This study does not involve more than minimal risk. We will protect participants and minimize risks by using the strict exclusion criteria and careful monitoring of adverse events (AEs). AEs will be tracked during regularly scheduled provider visits or through spontaneous reports made by participants. Drs. Ellerbeck, Salzman (or the treating provider) and Nollen will be made aware of unexpected or serious AEs within 24 hours of the first report by participants; all other AEs will be reviewed weekly by Dr. Nollen and discussed at regular meetings with Dr. Ellerbeck. SAEs will be reported to the KUMC IRB, FDA, and NIDA within 24 hours of first awareness of the event. Unexpected adverse events that are related to the study medication will be reported to KUMC IRB, FDA, and NIDA within 5 working days of first awareness of the event if the event is not serious fatal and within 24 hours of first awareness if the event is serious. Unexpected adverse events that are unrelated to the study medication will be reported to the KUMC HSC during yearly routine event reporting. IRB actions taken in this study will be reported to NIDA. Drs. Ellerbeck or Salzman (or the treating provider) will determine relatedness for each reported AE. SAEs will be defined as any event experienced by a study subject while on the study medication that is fatal, life-threatening (subject was at risk of death from the event as it occurred), disabling or incapacitating, requires inpatient hospitalization or prolongs a current hospitalization, is a congenital anomaly in the offspring of a subject who received the study medication, or required intervention to prevent permanent impairment or damage.

III. Subject Participation

A. Recruitment:

Participants will be recruited through clinic and community-based efforts. Flyers will be placed around Swope, KUMC and Truman Medical Center (TMC) for patients to take and providers will be asked to refer patients by providing them with the study line information. We will use the KUMC HERON database and the TMC electronic medical records to identify AA smokers and will ask their physician to send their patient a letter informing them of the study. We will also use the Frontiers registry to identify AA adult smokers who have agreed to be contacted for research. We will also use radio, TV, bus and Facebook ads and word of mouth, as needed, to recruit participants. Finally, AA smokers are currently being screened for other research studies being conducted by our team (i.e., active studies being conducted Dr. Nollen, Ellerbeck, or Cox). Those who are found to be ineligible for these studies will be informed about the current study and offered the opportunity to be screened. Recruitment letters, advertisements, and flyers are in the process of being developed. They will be submitted to the IRB for approval before any participants are enrolled.

B. Screening Interview/questionnaire

The screening interview will take place over the phone and be conducted by a member of the study team. The screening questionnaire will address the general inclusion/exclusion criteria in the Eligibility Criteria table above. Only participants who have express interest will be screened.

C. Informed consent process and timing of obtaining of consent

Consent will be obtained prior to participant involvement. Individuals interested in the proposed study will meet the research assistant at Swope or the CRU. Each individual will be given a copy of the consent form and as much time as they need to review its contents. After the consent form is read, both the individual and the research assistant will review the consent form together and the potential participant will be encouraged to ask questions. Each individual will be reminded that participation in the study is completely voluntary and their decision to participate will not affect their current or future medical care at the treating facility**.** The consenting process will take place in a private location.

D. Alternatives to Participation

Alternatives to participating in the study are to quit “cold turkey” (without assistance), use other smoking cessation programs, purchase other NRT from their pharmacy, obtain a prescription for varenicline, bupropion or other smoking cessation products from their physician, or continue to smoke.

E. Costs to Subjects

There are no costs to subjects. All tests, procedures, and visits are being performed solely for research purposes and are not billable to insurance companies.

F. How new information will be conveyed to the study subject and how it will be documented

We have plans to publish data from this study in aggregate but will not provide any individualized feedback to participants.

G. Payment, including a prorated plan for payment

Participants will be given a $30 electronically loaded gift card at Wks 0,2, 6 and 12, $20 gift card at Wk 18 and a $40 electronically loaded gift card at Wk 26 and a $10 electronically loaded gift card at Wks 1,3, 7 in appreciation for their time and participation. Participants may receive an additional $20 at Wks 2, 6, 12 and 18 for returning their unused medication. Participants must complete the visit to receive the reimbursement associated with that time point. Travel costs will not be reimbursed. Participants will also be given $20 for each referral who is eligible and enrolls in the study. Each participant may refer up to 3 total referrals.

H. Payment for a research-related injury

N/A

IV. Data Collection and Protection

A. Data Management and Security

Confidentiality will be maintained by assigning each participant a study identification number and numerically coding all data. The association of the ID-code and the participant's name will be kept by Tricia Snow in a locked file cabinet. The screening questionnaire and all survey data will be directly entered into RedCap or CRIS and accessible only by study staff. Any paper copies of records will be kept in a locked filing cabinet in offices that are kept locked when unoccupied. Only summaries of group data will be reported in any publications or presentations, with no identification of individuals. Because identifiable information will be collected, participant privacy will be maintained throughout the duration of the study by adhering to the regulations set forth by the HIPAA Privacy Rule. More specifically, identifiable information will not be released without written authorization of the participant. Mobile devices will not be used for data collection or storage. Identifiable data will not be sent outside of KUMC.

B. Sample / Specimen Collection

No more than 20 ml of urine will be collected at Weeks 0, 12, 18, and 26. Not more than 20ml of saliva will be collected at Week 0 for analysis of nicotine metabolism genotype and phenotype. All samples will be de-identified andlabeled with a study identification number. Samples will be stored at the Bioanalytical Laboratory at the Fairway Clinical Research Center under the direction of Greg Reed, PhD (urine), at the University of Toronto under the direction of Dr. Rachel Tyndale, PhD (urine and saliva), and at the University of California-San Francisco under the direction of Dr. Neal Benowitz, MD (urine and saliva). Samples will be accessible only to members the study team. Results from biomarker analyses will be de-identified and shared only with members of the research team. Any resulting publications will present the data in aggregate; individual participants will not be identified. Samples will be disposed of one month after the final report is sent out to the Principal Investigator, unless participants agree to have their urine stored for future testing. For participants who agree to future testing, samples will be stored indefinitely.

C. Tissue Banking Considerations

For participants who agree to future testing, samples will be stored indefinitely. New markers of nicotine and carcinogen exposure and genetic differences in nicotine metabolism are being discovered and the stored biological samples would be used for analysis of these new markers. All samples stored for future biomarker analyses will be de-identified and accessible only to members of the study team. Results from these analyses will be de-identified and shared only with members of the research team. Any resulting publications will present the data in aggregate; individual participants will not be identified.

D. Procedures to protect subject confidentiality

Confidentiality will be maintained by assigning each participant a study identification number and numerically coding all data. All biological samples and survey data will be labeled with the study identification number and never with the participants name or other identifiable information. The association of the ID-code and the participant's name will be kept by Tricia Snow in a locked file cabinet and will only be accessible to members of the study team.

E. Quality Assurance / Monitoring

All data will be directly entered into our electronic data capture system (i.e., RedCap or CRIS) that contains edit checks to control the quality and completeness of data entry. Completeness of data entry will be automatically verified before each assessment is completed. The electronic data

capture system is behind the KUMC secure firewall with role-based access that is HIPAA and human subjects compliant. There are no plans for ongoing third-party monitoring.

V. Data Analysis and Reporting

A. Statistical and Data Analysis

See II.C. above (Sample Size, Statistical Methods, and Power Calculations)

B. Outcome

See II.C. above (Sample Size, Statistical Methods, and Power Calculations)

C. Study results to participants

Study results will not be shared with participants.

D. Publication Plan

We plan to publish results in appropriate tobacco journals – e.g., JAMA, JNCI, Journal of General Internal Medicine, Addiction, Annals of Behavioral Medicine, Nicotine & Tobacco Research, Cancer Epidemiology, Biomarkers, & Prevention.

VI. Bibliography / References / Literature Cited

1. American Cancer Society. *Cancer Facts & Figures for African Americans, 2016-2018.* Atlanta, GA: American Cancer Society;2017.
2. Cunningham TJ, Croft JB, Liu Y, Lu H, Eke PI, Giles WH. Vital Signs: Racial Disparities in Age-Specific Mortality Among Blacks or African Americans - United States, 1999-2015. *MMWR Morb. Mortal. Wkly. Rep.* 2017;66(17):444-456.
3. Haiman CA, Stram DO, Wilkens LR, et al. Ethnic and racial differences in the smoking-related risk of lung cancer. *N. Engl. J. Med.* 2006;354(4):333-342.
4. Ahluwalia JS, McNagny SE, Clark WS. Smoking cessation among inner-city African Americans using the nicotine transdermal patch. *J. Gen. Intern. Med.* 1998;13(1):1-8.
5. Ahluwalia JS, Harris KJ, Catley D, Okuyemi KS, Mayo MS. Sustained-release bupropion for smoking cessation in African Americans: a randomized controlled trial. *JAMA.* 2002;288(4):468-474.
6. Cox LS, Okuyemi K, Choi WS, Ahluwalia JS. A Review of Tobacco Use Treatments in U.S. Ethnic Minority Populations. *Am. J. Health Promot.* 2011;25(5_suppl):S11-S30.
7. Cox LS, Nollen NL, Mayo MS, et al. Bupropion for smoking cessation in African American light smokers: a randomized controlled trial. *J. Natl. Cancer Inst.* 2012;104(4):290-298.
8. Robles GI, Singh-Franco D, Ghin HL. A review of the efficacy of smoking-cessation pharmacotherapies in nonwhite populations. *Clin. Ther.* 2008;30(5):800-812.
9. Webb MS. Treating tobacco dependence among African Americans: a meta-analytic review. *Health Psychol.* 2008;27(3s):S271-282.
10. Laude JR, Bailey SR, Crew E, et al. Extended treatment for cigarette smoking cessation: a randomized control trial. *Addiction.* 2017;112(8):1451-1459.
11. Fiore M, Jaen C, Baker T, et al. *Treating Tobacco Use and Dependence Clinical Practice Guideline: 2008 Update.* Washington, DC: U.S. Department of Health and Human Services; 2008.
12. Nollen NL, Cox LS, Yu Q, et al. A clinical trial to examine disparities in quitting between African-American and White adult smokers: Design, accrual, and baseline characteristics. *Contemp. Clin. Trials.* 2016;47:12-21.
13. 8. Pharmacologic Approaches to Glycemic Treatment. *Diabetes Care.* 2017;40(Supplement 1):S64-S74.
14. James PA, Oparil S, Carter BL, et al. 2014 evidence-based guideline for the management of high blood pressure in adults: Report from the panel members appointed to the eighth joint national committee (jnc 8). *JAMA.* 2014;311(5):507-520.

15. Rose JE, Behm FM. Adapting smoking cessation treatment according to initial response to precessation nicotine patch. *Am. J. Psychiatry.* 2013;170(8):860-867.
16. Baker TB, Mermelstein R, Collins LM, et al. New methods for tobacco dependence treatment research. *Ann. Behav. Med.* 2011;41(2):192-207.
17. Kidwell KM. SMART designs in cancer research: Past, present, and future. *Clin. Trials.* 2014;11(4):445-456.
18. U.S. Department of Health and Human Services. The Health Consequences of Smoking--50 Years of Progress. A Report of the Surgeon General. 2014;https://www.surgeongeneral.gov/library/reports/50-years-of-progress/.
19. American Cancer Society. *Cancer Facts & Figures for African Americans 2013-2014.* Atlanta, GA: American Cancer Society; 2013.
20. Babb S, Malarcher A, Schauer G, Asman K, Jamal A. Quitting Smoking Among Adults - United States, 2000-2015. *MMWR Morb. Mortal. Wkly. Rep.* 2017;65(52):1457-1464.
21. Croghan IT, Hurt RD, Ebbert JO, et al. Racial differences in smoking abstinence rates in a multicenter, randomized, open-label trial in the United States. *J Public Health.* 2010;18(1):59-68.
22. Cropsey KL, Weaver MF, Eldridge GD, Villalobos GC, Best AM, Stitzer ML. Differential success rates in racial groups: results of a clinical trial of smoking cessation among female prisoners. *Nicotine Tob Res.* 2009;11(6):690-697.
23. Gariti P, Lynch K, Alterman A, Kampman K, Xie H, Varillo K. Comparing smoking treatment programs for lighter smokers with and without a history of heavier smoking. *J. Subst. Abuse Treat.* 2009;37(3):247-255.
24. Murray RP, Connett JE, Buist AS, Gerald LB, Eichenhorn MS. Experience of Black participants in the Lung Health Study smoking cessation intervention program. *Nicotine Tob Res.* 2001;3(4):375-382.
25. Ahluwalia JS, Okuyemi K, Nollen N, et al. The effects of nicotine gum and counseling among African American light smokers: a 2 x 2 factorial design. *Addiction.* 2006;101(6):883-891.
26. Harris KJ, Ahluwalia JS, Okuyemi KS, et al. Addressing cultural sensitivity in a smoking cessation intervention: Development of the Kick it at Swope project. *Journal of Community Psycholoy.* 2001;29(4):447-458.
27. Jamal A, King BA, Neff LJ, Whitmill J, Babb SD, Graffunder CM. Current Cigarette Smoking Among Adults - United States, 2005-2015. *MMWR Morb. Mortal. Wkly. Rep.* 2016;65(44):1205-1211.
28. CDC. Coverage for Tobacco Use Cessation Treatments. National Center for Chronic Disease Prevention and Health Promotion, Office of Smoking and Health; 2014.
29. Bricker JB, Copeland W, Mull KE, et al. Single-arm trial of the second version of an acceptance & commitment therapy smartphone application for smoking cessation. *Drug Alcohol Depend.* 2017;170:37-42.
30. Bricker JB, Bush T, Zbikowski SM, Mercer LD, Heffner JL. Randomized trial of telephone-delivered acceptance and commitment therapy versus cognitive behavioral therapy for smoking cessation: a pilot study. *Nicotine Tob Res.* 2014;16(11):1446-1454.
31. Ciccolo JT, Williams DM, Dunsiger SI, et al. Efficacy of Resistance Training as an Aid to Smoking Cessation: Rationale and Design of the Strength To Quit Study. *Mental health and physical activity.* 2014;7(2):95-103.
32. Gonzales D, Rennard SI, Nides M, et al. Varenicline, an alpha4beta2 nicotinic acetylcholine receptor partial agonist, vs sustained-release bupropion and placebo for smoking cessation: a randomized controlled trial. *JAMA.* 2006;296(1):47-55.
33. Jorenby DE, Hays JT, Rigotti NA, et al. Efficacy of varenicline, an alpha4beta2 nicotinic acetylcholine receptor partial agonist, vs placebo or sustained-release bupropion for smoking cessation: a randomized controlled trial. *JAMA.* 2006;296(1):56-63.
34. Jorenby DE, Leischow SJ, Nides MA, et al. A controlled trial of sustained-release bupropion, a nicotine patch, or both for smoking cessation. *N. Engl. J. Med.* 1999;340(9):685-691.
35. Malcolm RE, Sillett RW, Turner JA, Ball KP. The use of nicotine chewing gum as an aid to stopping smoking. *Psychopharmacology (Berl.).* 1980;70(3):295-296.
36. Ferguson SG, Gitchell JG, Shiffman S, Sembower MA. Prediction of abstinence at 10 weeks based on smoking status at 2 weeks during a quit attempt: secondary analysis of two parallel, 10-week, randomized, double-blind, placebo-controlled clinical trials of 21-mg nicotine patch in adult smokers. *Clin. Ther.* 2009;31(9):1957-1965.
37. Jamerson BD, Nides M, Jorenby DE, et al. Late-term smoking cessation despite initial failure: an evaluation of bupropion sustained release, nicotine patch, combination therapy, and placebo. *Clin. Ther.* 2001;23(5):744-752.
38. Kenford SL, Fiore MC, Jorenby DE, Smith SS, Wetter D, Baker TB. Predicting smoking cessation. Who will quit with and without the nicotine patch. *JAMA.* 1994;271(8):589-594.
39. Jorenby DE, Leischow SJ, Nides MA, et al. A Controlled Trial of Sustained-Release Bupropion, a Nicotine Patch, or Both for Smoking Cessation. *N. Engl. J. Med.* 1999;340(9):685-691.
40. Durcan MJ, White J, Jorenby DE, et al. Impact of prior nicotine replacement therapy on smoking cessation efficacy. *Am. J. Health Behav.* 2002;26(3):213-220.
41. Ellerbeck EF, Mahnken JD, Cupertino AP, et al. Effect of varying levels of disease management on smoking cessation: a randomized trial. *Ann. Intern. Med.* 2009;150(7):437-446.
42. Gonzales D, Hajek P, Pliamm L, et al. Retreatment with varenicline for smoking cessation in smokers who have previously taken varenicline: a randomized, placebo-controlled trial. *Clin. Pharmacol. Ther.* 2014;96(3):390-396.

43. Gonzales DH, Nides MA, Ferry LH, et al. Bupropion SR as an aid to smoking cessation in smokers treated previously with bupropion: a randomized placebo-controlled study. *Clin. Pharmacol. Ther.* 2001;69(6):438-444.
44. Gourlay SG, Forbes A, Marriner T, Pethica D, McNeil JJ. Double blind trial of repeated treatment with transdermal nicotine for relapsed smokers. *BMJ.* 1995;311(7001):363-366.
45. Hajek P, Stead LF, West R, Jarvis M, Hartmann-Boyce J, Lancaster T. Relapse prevention interventions for smoking cessation. *Cochrane Database Syst Rev.* 2013(8):CD003999.
46. Shiffman S, Dresler CM, Rohay JM. Successful treatment with a nicotine lozenge of smokers with prior failure in pharmacological therapy. *Addiction.* 2004;99(1):83-92.
47. Tonnesen P, Mikkelsen K, Norregaard J, Jorgensen S. Recycling of hard-core smokers with nicotine nasal spray. *Eur. Respir. J.* 1996;9(8):1619-1623.
48. Joseph AM, Rice K, An LC, Mohiuddin A, Lando H. Recent quitters' interest in recycling and harm reduction. *Nicotine Tob Res.* 2004;6(6):1075-1077.
49. Ebbert JO, Little MA, Klesges RC, et al. Step Care treatment for smoking cessation. *Health Educ. Res.* 2017;32(1):1-11.
50. Almirall D, Nahum-Shani I, Sherwood NE, Murphy SA. Introduction to SMART designs for the development of adaptive interventions: with application to weight loss research. *Transl. Behav. Med.* 2014;4(3):260-274.
51. Almirall D, Compton SN, Rynn MA, Walkup JT, Murphy SA. SMARTer discontinuation trial designs for developing an adaptive treatment strategy. *J. Child Adolesc. Psychopharmacol.* 2012;22(5):364-374.
52. Dawson R, Lavori PW. Placebo-free designs for evaluating new mental health treatments: the use of adaptive treatment strategies. *Stat. Med.* 2004;23(21):3249-3262.
53. Wang L, Rotnitzky A, Lin X, Millikan RE, Thall PF. Evaluation of Viable Dynamic Treatment Regimes in a Sequentially Randomized Trial of Advanced Prostate Cancer. *J Am Stat Assoc.* 2012;107(498):493-508.
54. Cook JW, Collins LM, Fiore MC, et al. Comparative effectiveness of motivation phase intervention components for use with smokers unwilling to quit: a factorial screening experiment. *Addiction.* 2016;111(1):117-128.
55. Piper ME, Fiore MC, Smith SS, et al. Identifying effective intervention components for smoking cessation: a factorial screening experiment. *Addiction.* 2016;111(1):129-141.
56. Rose JE, Behm FM. Combination treatment with varenicline and bupropion in an adaptive smoking cessation paradigm. *Am. J. Psychiatry.* 2014;171(11):1199-1205.
57. Schlam TR, Fiore MC, Smith SS, et al. Comparative effectiveness of intervention components for producing long-term abstinence from smoking: a factorial screening experiment. *Addiction.* 2016;111(1):142-155.
58. Heckman BW, Cummings KM, Kasza KA, et al. Effectiveness of Switching Smoking-Cessation Medications Following Relapse. *Am J Prev Med.* 2017;53(2):e63-e70.
59. Caraballo RS, Yee SL, Gfroerer J, Mirza SA. Adult tobacco use among racial and ethnic groups living in the United States, 2002-2005. *Prev. Chronic Dis.* 2008;5(3):A78.
60. Caraballo RS, Giovino GA, Pechacek TF, et al. Racial and ethnic differences in serum cotinine levels of cigarette smokers: Third National Health and Nutrition Examination Survey, 1988-1991. *JAMA.* 1998;280(2):135-139.
61. Lynam I, Catley D, Harris KJ, Goggin K, Berkley-Patton J, Thomas J. African American smokers' intention to use pharmacotherapy for cessation. *Am. J. Health Behav.* 2012;36(5):615-627.
62. Faseru B, Nollen NL, Mayo MS, et al. Predictors of cessation in African American light smokers enrolled in a bupropion clinical trial. *Addict. Behav.* 2013;38(3):1796-1803.
63. Harris KJ, Okuyemi KS, Catley D, Mayo MS, Ge B, Ahluwalia JS. Predictors of smoking cessation among African-Americans enrolled in a randomized controlled trial of bupropion. *Prev. Med.* 2004;38(4):498-502.
64. Nollen NL, Mayo MS, Sanderson Cox L, et al. Predictors of quitting among African American light smokers enrolled in a randomized, placebo-controlled trial. *J. Gen. Intern. Med.* 2006;21(6):590-595.
65. Nollen NL, Mayo MS, Ahluwalia JS, et al. Factors associated with discontinuation of bupropion and counseling among African American light smokers in a randomized clinical trial. *Ann. Behav. Med.* 2013;46(3):336-348.
66. Zhu AZ, Cox LS, Nollen N, et al. CYP2B6 and bupropion's smoking-cessation pharmacology: the role of hydroxybupropion. *Clin. Pharmacol. Ther.* 2012;92(6):771-777.
67. Bolt DM, Piper ME, Theobald WE, Baker TB. Why Two Smoking Cessation Agents Work Better than One: Role of Craving Suppression. *J. Consult. Clin. Psychol.* 2012;80(1):54-65.
68. Ferguson SG, Shiffman S, Gwaltney CJ. Does reducing withdrawal severity mediate nicotine patch efficacy? A randomized clinical trial. *J. Consult. Clin. Psychol.* 2006;74(6):1153-1161.
69. Lerman C, Roth D, Kaufmann V, et al. Mediating mechanisms for the impact of bupropion in smoking cessation treatment. *Drug Alcohol Depend.* 2002;67(2):219-223.
70. McCarthy DE, Piasecki TM, Lawrence DL, Jorenby DE, Shiffman S, Baker TB. Psychological mediators of bupropion sustained-release treatment for smoking cessation. *Addiction.* 2008;103(9):1521-1533.
71. Piper ME, Federmen EB, McCarthy DE, et al. Using mediational models to explore the nature of tobacco motivation and tobacco treatment effects. *J. Abnorm. Psychol.* 2008;117(1):94-105.

72. Stanton CA, Lloyd-Richardson EE, Papandonatos GD, de Dios MA, Niaura R. Mediators of the relationship between nicotine replacement therapy and smoking abstinence among people living with HIV/AIDS. *AIDS Educ. Prev.* 2009;21(3 Suppl):65-80.
73. Siahpush M, Yong HH, Borland R, Reid JL, Hammond D. Smokers with financial stress are more likely to want to quit but less likely to try or succeed: findings from the International Tobacco Control (ITC) Four Country Survey. *Addiction.* 2009;104(8):1382-1390.
74. Hughes JR. An updated algorithm for choosing among smoking cessation treatments. *J. Subst. Abuse Treat.*;45(2):215-221.
75. Lerman C, Schnoll RA, Hawk LW, Jr., et al. Use of the nicotine metabolite ratio as a genetically informed biomarker of response to nicotine patch or varenicline for smoking cessation: a randomised, double-blind placebo-controlled trial. *The Lancet. Respiratory medicine.* 2015;3(2):131-138.
76. Schnoll RA, Patterson F, Wileyto EP, et al. Effectiveness of extended-duration transdermal nicotine therapy: a randomized trial. *Ann. Intern. Med.* 2010;152(3):144-151.
77. Webb Hooper M, Antoni MH, Okuyemi K, Dietz NA, Resnicow K. Randomized Controlled Trial of Group-Based Culturally Specific Cognitive Behavioral Therapy Among African American Smokers. *Nicotine Tob Res.* 2017;19(3):333-341.
78. Fu SS, Burgess D, van Ryn M, Hatsukami DK, Solomon J, Joseph AM. Views on smoking cessation methods in ethnic minority communities: a qualitative investigation. *Prev. Med.* 2007;44(3):235-240.
79. Zyban® (bupropion hydrochloride) sustained-release tablets: product information. 2016; https://www.gsksource.com/pharma/content/dam/GlaxoSmithKline/US/en/Prescribing_Information/Zyban/pdf/ZYBAN-PI-MG.PDF. Accessed October 19, 2016.
80. Hajek P, McRobbie H, Myers Smith K, Phillips A, Cornwall D, Dhanji A. Increasing varenicline dose in smokers who do not respond to the standard dosage: A randomized clinical trial. *JAMA Internal Medicine.* 2015;175(2):266-271.
81. Hatsukami D, Mooney M, Murphy S, LeSage M, Babb D, Hecht S. Effects of High Dose Transdermal Nicotine Replacement in Cigarette Smokers. *Pharmacology, biochemistry, and behavior.* 2007;86(1):132-139.
82. Ebbert JO, Hatsukami DK, Croghan IT, et al. Combination varenicline and bupropion SR for tobacco-dependence treatment in cigarette smokers: a randomized trial. *JAMA.* 2014;311(2):155-163.
83. Koegelenberg CF, Noor F, Bateman ED, et al. Efficacy of varenicline combined with nicotine replacement therapy vs varenicline alone for smoking cessation: a randomized clinical trial. *JAMA.* 2014;312(2):155-161.
84. Piper ME, Smith SS, Schlam TR, et al. A randomized placebo-controlled clinical trial of five smoking cessation pharmacotherapies. *Arch. Gen. Psychiatry.* 2009;66(11):1253-1262.
85. Stapleton J, West R, Hajek P, et al. Randomized trial of nicotine replacement therapy (NRT), bupropion and NRT plus bupropion for smoking cessation: effectiveness in clinical practice. *Addiction.* 2013;108(12):2193-2201.
86. Hughes JR, Keely JP, Niaura RS, Ossip-Klein DJ, Richmond RL, Swan GE. Measures of abstinence in clinical trials: issues and recommendations. *Nicotine Tob Res.* 2003;5(1):13-25.
87. Jacob P, 3rd, Hatsukami D, Severson H, Hall S, Yu L, Benowitz NL. Anabasine and anatabine as biomarkers for tobacco use during nicotine replacement therapy. *Cancer Epidemiol. Biomarkers Prev.* 2002;11(12):1668-1673.
88. Marrone GF, Shakleya DM, Scheidweiler KB, Singleton EG, Huestis MA, Heishman SJ. Relative performance of common biochemical indicators in detecting cigarette smoking. *Addiction.* 2011;106(7):1325-1334.
89. Klein K, Lang T, Saussele T, et al. Genetic variability of CYP2B6 in populations of African and Asian origin: allele frequencies, novel functional variants, and possible implications for anti-HIV therapy with efavirenz. *Pharmacogenet. Genomics.* 2005;15(12):861-873.
90. Okuyemi KS, Zheng H, Guo H, Ahluwalia JS. Predictors of adherence to nicotine gum and counseling among African-American light smokers. *J. Gen. Intern. Med.* 2010;25(9):969-976.
91. Shiffman S, Rolf CN, Hellebusch SJ, et al. Real-world efficacy of prescription and over-the-counter nicotine replacement therapy. *Addiction.* 2002;97(5):505-516.
92. Trinidad DR, Perez-Stable EJ, Emery SL, White MM, Grana RA, Messer KS. Intermittent and light daily smoking across racial/ethnic groups in the United States. *Nicotine Tob Res.* 2009;11(2):203-210.
93. Hurt RD, Ebbert JO, Hays JT, McFadden DD. Treating tobacco dependence in a medical setting. *CA Cancer J. Clin.* 2009;59(5):314-326.
94. Krupski L, Cummings KM, Hyland A, Carlin-Menter S, Toll BA, Mahoney MC. Nicotine replacement therapy distribution to light daily smokers calling a quitline. *Nicotine Tob Res.* 2013;15(9):1572-1577.
95. Shiffman S. Nicotine lozenge efficacy in light smokers. *Drug Alcohol Depend.* 2005;77(3):311-314.
96. Clinical Practice Guideline Treating Tobacco U, Dependence Update Panel L, Staff. A clinical practice guideline for treating tobacco use and dependence: 2008 update. A U.S. Public Health Service report. *Am. J. Prev. Med.* 2008;35(2):158-176.
97. Perkins KA, Conklin CA, Levine MD. *Cognitive-behavioral therapy for smoking cessation: a practical guidebook to the most effective treatments.* Taylor & Francis; 2008.

98. Catley D, Goggin K, Harris KJ, et al. A Randomized Trial of Motivational Interviewing: Cessation Induction Among Smokers With Low Desire to Quit. *Am. J. Prev. Med.* 2016;50(5):573-583.
99. Toll BA, O'Malley SS, McKee SA, Salovey P, Krishnan-Sarin S. Confirmatory Factor Analysis of the Minnesota Nicotine Withdrawal Scale. *Psychology of addictive behaviors : journal of the Society of Psychologists in Addictive Behaviors.* 2007;21(2):216-225.
100. Cox LS, Tiffany ST, Christen AG. Evaluation of the brief questionnaire of smoking urges (QSU-brief) in laboratory and clinical settings. *Nicotine Tob Res.* 2001;3(1):7-16.
101. Cappelleri JC, Bushmakin AG, Baker CL, Merikle E, Olufade AO, Gilbert DG. Confirmatory factor analyses and reliability of the modified cigarette evaluation questionnaire. *Addict. Behav.* 2007;32(5):912-923.
102. Watson D, Clark LA, Tellegen A. Development and validation of brief measures of positive and negative affect: the PANAS scales. *J. Pers. Soc. Psychol.* 1988;54(6):1063-1070.
103. Etter JF, Bergman MM, Humair JP, Perneger TV. Development and validation of a scale measuring self-efficacy of current and former smokers. *Addiction.* 2000;95(6):901-913.
104. Nollen NL, Cox LS, Nazir N, et al. A pilot clinical trial of varenicline for smoking cessation in black smokers. *Nicotine Tob Res.* 2011;13(9):868-873.
105. Schnoll RA, Patterson F, Wileyto EP, Tyndale RF, Benowitz N, Lerman C. Nicotine metabolic rate predicts successful smoking cessation with transdermal nicotine: a validation study. *Pharmacol. Biochem. Behav.* 2009;92(1):6-11.
106. Brown RA, Burgess E, Sales SD, Whiteley JA, Evans DMM, I.W. Reliability and validity of a smoking timeline follow-back interview. *Psychology of Addictive Behavior.* 1998;12(2):101-112.
107. Buchanan TS, Berg CJ, Cox LS, et al. Adherence to varenicline among African American smokers: an exploratory analysis comparing plasma concentration, pill count, and self-report. *Nicotine Tob Res.* 2012;14(9):1083-1091.
108. Fagerstrom K, Russ C, Yu CR, Yunis C, Foulds J. The Fagerstrom Test for Nicotine Dependence as a predictor of smoking abstinence: a pooled analysis of varenicline clinical trial data. *Nicotine Tob Res.* 2012;14(12):1467-1473.
109. Al-Delaimy WK, Edland S, Pierce JP, Mills AL, White M. *Technical Report on Analytic Methods and Approaches Used in the 2008 California Tobacco Survey Analysis. Vol 1: Data Collection Methodology.* La Jolla, CA: University of California, San Diego;2009.
110. Centers for Disease Control and Prevention. *Behavioral Risk Factor Surveillance System Survey Questionnaire.* Atlanta, Georgia: U.S. Department of Health and Human Services,

Centers for Disease Control and Prevention;2012.
111. Centers for Disease Control and Prevention (CDC), National Center for Health Statistics (NCHS). *National Health and Nutrition Examination Survey.* Hyattsville, MD: U.S. Department of Health and Human Services, Centers for Disease Control and Prevention;2012.
112. Dorsey R, Graham G. New HHS data standards for race, ethnicity, sex, primary language, and disability status. *JAMA.* 2011;306(21):2378-2379.
113. Kroenke K, Spitzer RL, Williams JB. The Patient Health Questionnaire-2: validity of a two-item depression screener. *Med. Care.* 2003;41(11):1284-1292.
114. Spitzer RL, Kroenke K, Williams JB, Lowe B. A brief measure for assessing generalized anxiety disorder: the GAD-7. *Arch. Intern. Med.* 2006;166(10):1092-1097.
115. Lerman C, Schnoll RA, Hawk LW, et al. A Randomized Placebo-controlled Trial to Test a Genetically-informed Biomarker ForPersonalizing Treatment for Tobacco Dependence. *The Lancet. Respiratory medicine.* 2015;3(2):131-138.
116. Benowitz N, Jacob P, Ahijevych K, et al. Biochemical verification of tobacco use and cessation. *Nicotine Tob Res.* 2002;4(2):149-159.
117. Kotz D, West R. Explaining the social gradient in smoking cessation: it's not in the trying, but in the succeeding. *Tob. Control.* 2009;18(1):43-46.
118. Piper ME, Cook JW, Schlam TR, et al. Gender, race, and education differences in abstinence rates among participants in two randomized smoking cessation trials. *Nicotine Tob Res.* 2010;12(6):647-657.
119. Wetter DW, Kenford SL, Smith SS, Fiore MC, Jorenby DE, Baker TB. Gender differences in smoking cessation. *J. Consult. Clin. Psychol.* 1999;67(4):555-562.
120. Baron RM, Kenny DA. The moderator-mediator variable distinction in social psychological research: conceptual, strategic, and statistical considerations. *J. Pers. Soc. Psychol.* 1986;51(6):1173-1182.

APPENDIX I: VULNERABLE POPULATIONS

Not Applicable

1. U.S. Department of Health and Human Services. The Health Consequences of Smoking--50 Years of Progress. A Report of the Surgeon General. 2014;https://www.surgeongeneral.gov/library/reports/50-years-of-progress/.
2. American Cancer Society. *Cancer Facts & Figures for African Americans 2013-2014.* Atlanta, GA: American Cancer Society; 2013.
3. American Cancer Society. *Cancer Facts & Figures for African Americans, 2016-2018.* Atlanta, GA: American Cancer Society;2017.
4. Cunningham TJ, Croft JB, Liu Y, Lu H, Eke PI, Giles WH. Vital Signs: Racial Disparities in Age-Specific Mortality Among Blacks or African Americans - United States, 1999-2015. *MMWR Morb. Mortal. Wkly. Rep.* 2017;66(17):444-456.
5. Haiman CA, Stram DO, Wilkens LR, et al. Ethnic and racial differences in the smoking-related risk of lung cancer. *N. Engl. J. Med.* 2006;354(4):333-342.
6. Fiore M, Jaen C, Baker T, et al. *Treating Tobacco Use and Dependence Clinical Practice Guideline: 2008 Update.* Washington, DC: U.S. Department of Health and Human Services; 2008.
7. Babb S, Malarcher A, Schauer G, Asman K, Jamal A. Quitting Smoking Among Adults - United States, 2000-2015. *MMWR Morb. Mortal. Wkly. Rep.* 2017;65(52):1457-1464.
8. Croghan IT, Hurt RD, Ebbert JO, et al. Racial differences in smoking abstinence rates in a multicenter, randomized, open-label trial in the United States. *J Public Health.* 2010;18(1):59-68.
9. Cropsey KL, Weaver MF, Eldridge GD, Villalobos GC, Best AM, Stitzer ML. Differential success rates in racial groups: results of a clinical trial of smoking cessation among female prisoners. *Nicotine Tob Res.* 2009;11(6):690-697.
10. Gariti P, Lynch K, Alterman A, Kampman K, Xie H, Varillo K. Comparing smoking treatment programs for lighter smokers with and without a history of heavier smoking. *J. Subst. Abuse Treat.* 2009;37(3):247-255.
11. Murray RP, Connett JE, Buist AS, Gerald LB, Eichenhorn MS. Experience of Black participants in the Lung Health Study smoking cessation intervention program. *Nicotine Tob Res.* 2001;3(4):375-382.
12. Nollen NL, Cox LS, Yu Q, et al. A clinical trial to examine disparities in quitting between African-American and White adult smokers: Design, accrual, and baseline characteristics. *Contemp. Clin. Trials.* 2016;47:12-21.
13. Ahluwalia JS, McNagny SE, Clark WS. Smoking cessation among inner-city African Americans using the nicotine transdermal patch. *J. Gen. Intern. Med.* 1998;13(1):1-8.
14. Ahluwalia JS, Harris KJ, Catley D, Okuyemi KS, Mayo MS. Sustained-release bupropion for smoking cessation in African Americans: a randomized controlled trial. *JAMA.* 2002;288(4):468-474.
15. Cox LS, Nollen NL, Mayo MS, et al. Bupropion for smoking cessation in African American light smokers: a randomized controlled trial. *J. Natl. Cancer Inst.* 2012;104(4):290-298.
16. Cox LS, Okuyemi K, Choi WS, Ahluwalia JS. A Review of Tobacco Use Treatments in U.S. Ethnic Minority Populations. *Am. J. Health Promot.* 2011;25(5_suppl):S11-S30.
17. Webb MS. Treating tobacco dependence among African Americans: a meta-analytic review. *Health Psychol.* 2008;27(3s):S271-282.
18. Robles GI, Singh-Franco D, Ghin HL. A review of the efficacy of smoking-cessation pharmacotherapies in nonwhite populations. *Clin. Ther.* 2008;30(5):800-812.
19. Ahluwalia JS, Okuyemi K, Nollen N, et al. The effects of nicotine gum and counseling among African American light smokers: a 2 x 2 factorial design. *Addiction.* 2006;101(6):883-891.
20. Harris KJ, Ahluwalia JS, Okuyemi KS, et al. Addressing cultural sensitivity in a smoking cessation intervention: Development of the Kick it at Swope project. *Journal of Community Psycholoy.* 2001;29(4):447-458.
21. Gonzales D, Rennard SI, Nides M, et al. Varenicline, an alpha4beta2 nicotinic acetylcholine receptor partial agonist, vs sustained-release bupropion and placebo for smoking cessation: a randomized controlled trial. *JAMA.* 2006;296(1):47-55.
22. Jorenby DE, Hays JT, Rigotti NA, et al. Efficacy of varenicline, an alpha4beta2 nicotinic acetylcholine receptor partial agonist, vs placebo or sustained-release bupropion for smoking cessation: a randomized controlled trial. *JAMA.* 2006;296(1):56-63.
23. Jorenby DE, Leischow SJ, Nides MA, et al. A controlled trial of sustained-release bupropion, a nicotine patch, or both for smoking cessation. *N. Engl. J. Med.* 1999;340(9):685-691.
24. Malcolm RE, Sillett RW, Turner JA, Ball KP. The use of nicotine chewing gum as an aid to stopping smoking. *Psychopharmacology (Berl.).* 1980;70(3):295-296.
25. Ferguson SG, Gitchell JG, Shiffman S, Sembower MA. Prediction of abstinence at 10 weeks based on smoking status at 2 weeks during a quit attempt: secondary analysis of two parallel, 10-week, randomized, double-blind, placebo-controlled clinical trials of 21-mg nicotine patch in adult smokers. *Clin. Ther.* 2009;31(9):1957-1965.

26. Jamerson BD, Nides M, Jorenby DE, et al. Late-term smoking cessation despite initial failure: an evaluation of bupropion sustained release, nicotine patch, combination therapy, and placebo. *Clin. Ther.* 2001;23(5):744-752.
27. Kenford SL, Fiore MC, Jorenby DE, Smith SS, Wetter D, Baker TB. Predicting smoking cessation. Who will quit with and without the nicotine patch. *JAMA.* 1994;271(8):589-594.
28. Jorenby DE, Leischow SJ, Nides MA, et al. A Controlled Trial of Sustained-Release Bupropion, a Nicotine Patch, or Both for Smoking Cessation. *N. Engl. J. Med.* 1999;340(9):685-691.
29. Hajek P, Stead LF, West R, Jarvis M, Hartmann-Boyce J, Lancaster T. Relapse prevention interventions for smoking cessation. *Cochrane Database Syst Rev.* 2013(8):CD003999.
30. Baker TB, Collins LM, Mermelstein R, et al. Enhancing the effectiveness of smoking treatment research: conceptual bases and progress. *Addiction.* 2016;111(1):107-116.
31. Baker TB, Mermelstein R, Collins LM, et al. New methods for tobacco dependence treatment research. *Ann. Behav. Med.* 2011;41(2):192-207.
32. Almirall D, Nahum-Shani I, Sherwood NE, Murphy SA. Introduction to SMART designs for the development of adaptive interventions: with application to weight loss research. *Transl. Behav. Med.* 2014;4(3):260-274.
33. Almirall D, Compton SN, Rynn MA, Walkup JT, Murphy SA. SMARTer discontinuation trial designs for developing an adaptive treatment strategy. *J. Child Adolesc. Psychopharmacol.* 2012;22(5):364-374.
34. Dawson R, Lavori PW. Placebo-free designs for evaluating new mental health treatments: the use of adaptive treatment strategies. *Stat. Med.* 2004;23(21):3249-3262.
35. Kidwell KM. SMART designs in cancer research: Past, present, and future. *Clin. Trials.* 2014;11(4):445-456.
36. Wang L, Rotnitzky A, Lin X, Millikan RE, Thall PF. Evaluation of Viable Dynamic Treatment Regimes in a Sequentially Randomized Trial of Advanced Prostate Cancer. *J Am Stat Assoc.* 2012;107(498):493-508.
37. James PA, Oparil S, Carter BL, et al. 2014 evidence-based guideline for the management of high blood pressure in adults: Report from the panel members appointed to the eighth joint national committee (jnc 8). *JAMA.* 2014;311(5):507-520.
38. 8. Pharmacologic Approaches to Glycemic Treatment. *Diabetes Care.* 2017;40(Supplement 1):S64-S74.
39. Cook JW, Collins LM, Fiore MC, et al. Comparative effectiveness of motivation phase intervention components for use with smokers unwilling to quit: a factorial screening experiment. *Addiction.* 2016;111(1):117-128.
40. Piper ME, Fiore MC, Smith SS, et al. Identifying effective intervention components for smoking cessation: a factorial screening experiment. *Addiction.* 2016;111(1):129-141.
41. Rose JE, Behm FM. Adapting smoking cessation treatment according to initial response to precessation nicotine patch. *Am. J. Psychiatry.* 2013;170(8):860-867.
42. Rose JE, Behm FM. Combination treatment with varenicline and bupropion in an adaptive smoking cessation paradigm. *Am. J. Psychiatry.* 2014;171(11):1199-1205.
43. Schlam TR, Fiore MC, Smith SS, et al. Comparative effectiveness of intervention components for producing long-term abstinence from smoking: a factorial screening experiment. *Addiction.* 2016;111(1):142-155.
44. Laude JR, Bailey SR, Crew E, et al. Extended treatment for cigarette smoking cessation: a randomized control trial. *Addiction.* 2017;112(8):1451-1459.
45. Heckman BW, Cummings KM, Kasza KA, et al. Effectiveness of Switching Smoking-Cessation Medications Following Relapse. *Am. J. Prev. Med.* 2017;53(2):e63-e70.
46. Caraballo RS, Yee SL, Gfroerer J, Mirza SA. Adult tobacco use among racial and ethnic groups living in the United States, 2002-2005. *Prev. Chronic Dis.* 2008;5(3):A78.
47. Caraballo RS, Giovino GA, Pechacek TF, et al. Racial and ethnic differences in serum cotinine levels of cigarette smokers: Third National Health and Nutrition Examination Survey, 1988-1991. *JAMA.* 1998;280(2):135-139.
48. Lynam I, Catley D, Harris KJ, Goggin K, Berkley-Patton J, Thomas J. African American smokers' intention to use pharmacotherapy for cessation. *Am. J. Health Behav.* 2012;36(5):615-627.
49. Hurt RD, Ebbert JO, Hays JT, McFadden DD. Treating tobacco dependence in a medical setting. *CA Cancer J. Clin.* 2009;59(5):314-326.
50. Clinical Practice Guideline Treating Tobacco U, Dependence Update Panel L, Staff. A clinical practice guideline for treating tobacco use and dependence: 2008 update. A U.S. Public Health Service report. *Am. J. Prev. Med.* 2008;35(2):158-176.
51. Perkins KA, Conklin CA, Levine MD. *Cognitive-behavioral therapy for smoking cessation: a practical guidebook to the most effective treatments.* Taylor & Francis; 2008.
52. Webb Hooper M, Antoni MH, Okuyemi K, Dietz NA, Resnicow K. Randomized Controlled Trial of Group-Based Culturally Specific Cognitive Behavioral Therapy Among African American Smokers. *Nicotine Tob Res.* 2017;19(3):333-341.
53. Catley D, Goggin K, Harris KJ, et al. A Randomized Trial of Motivational Interviewing: Cessation Induction Among Smokers With Low Desire to Quit. *Am. J. Prev. Med.* 2016;50(5):573-583.

54. Anthenelli RM, Benowitz NL, West R, et al. Neuropsychiatric safety and efficacy of varenicline, bupropion, and nicotine patch in smokers with and without psychiatric disorders (EAGLES): a double-blind, randomised, placebo-controlled clinical trial. *Lancet.* 2016;387(10037):2507-2520.
