# Supplementary material for: Early Treatment Response in Black Smokers Undergoing Pharmacotherapy for Smoking Cessation: A Secondary Analysis of a Randomized Clinical Trial
Source: JAMA Netw Open. 2023 Sep 20;6(9):e2334695. doi: 10.1001/jamanetworkopen.2023.34695 (PMC10512105; doi:10.1001/jamanetworkopen.2023.34695)
Supplement: Supplement 2. — eFigure. Study Flowchart [file jamanetwopen-e2334695-s002.pdf]

# Supplemental Online Content

Leavens ELS, Mayo MS, Brown AR, et al. Early treatment response in Black smokers undergoing pharmacotherapy for smoking cessation: a secondary analysis of a randomized clinical trial. *JAMA Netw Open*. 2023;6(9):e2334695. doi:10.1001/jamanetworkopen.2023.34695

**eFigure.** Study Flowchart

This supplemental material has been provided by the authors to give readers additional information about their work.

© 2023 Leavens ELS et al. *JAMA Network Open.*

893 Black adults completed initial phone screening

143 excluded after demographic and tobacco use portions
- 73 used other tobacco products
- 43 did not smoke 5-30 cigarettes per day
- 29 did not smoke 25 or more days per month
- 28 problem drinking (binge and/or unwilling to limit alcohol)
- 25 used cessation pharmacotherapy or e-cigs in past 30 days
- 14 not non-Hispanic Black
- 12 lived in shelter or on the street
- 11 did not smoke at current rate for 6 months or longer
- 7 unwilling to take study medication
- 5 not staying in the area for 6 months
- 5 unwilling to use birth control to avoid pregnancy
- 3 pregnant, planning to get pregnant, or breast feeding
- 3 another household member enrolled
- 2 unable to make study visits
- 1 unwilling to refrain from non-study provided pharmacotherapy
- 1 unwilling to set a quit date
- 1 not 18 years or older

124 excluded after medical screening
- 65 used select medication(s)
- 34 endorsed select psychiatric condition(s)
- 33 endorsed select medical condition(s)
- 7 did not receive medical authorization/clearance by provider
- 6 alcohol or drug treatment in past year
- 6 previous adverse reaction to study medications
- 1 refused medical authorization/clearance

626 cleared to take the study medication and scheduled for final, in-person screening

229 did not return

397 attended final, in-person screening

5 medically ineligible at in person screening
- 3 CO < 5 ppm (nonsmoker)
- 2 declined to sign informed consent
- 1 positive pregnancy test or refused

392 randomized

196 randomized to OPT

187 (95.4%) completed week 2 study visit
173 (88.3%) completed week 6 study visit
165 (84.2%) completed week 12 study visit
163 (83.2%) completed week 18 study visit
162 (82.7%) completed week 26 study visit

187 included in primary analysis

196 randomized to UC

187 (95.4%) completed week 2 study visit
178 (90.8%) completed week 6 study visit
174 (88.8%) completed week 12 study visit
170 (86.7%) completed week 18 study visit
162 (82.7%) completed week 26 study visit

187 included in primary analysis

© 2023 Leavens ELS et al. *JAMA Network Open.*
