# Supplementary material for: Early Treatment Response in Black Smokers Undergoing Pharmacotherapy for Smoking Cessation: A Secondary Analysis of a Randomized Clinical Trial
Source: JAMA Netw Open. 2023 Sep 20;6(9):e2334695. doi: 10.1001/jamanetworkopen.2023.34695 (PMC10512105; doi:10.1001/jamanetworkopen.2023.34695)
Supplement: Supplement 3. — Data Sharing Statement [file jamanetwopen-e2334695-s003.pdf]

Data Sharing Statement

Leavens. Early Treatment Response in Black Smokers Undergoing Pharmacotherapy for Smoking Cessation: A Secondary Analysis of a Randomized Clinical Trial. *JAMA Netw Open*. Published online September 20, 2023. doi:10.1001/jamanetworkopen.2023.34695

Data
Data available: No

Additional Information
Explanation for why data not available: The data that support the findings of this study are available from the corresponding author, [ELSL], upon reasonable request.
